# Extracellular Vesicle miR-558 Regulates Endothelial Function Through HMGB2 in Adult Moyamoya Disease

**DOI:** 10.3390/cells15161456

**Published:** 2026-08-13

**Authors:** Eun Hee Kim, Oh Young Bang, Gyun Sik Oh, Mi Jeong Oh, Woo Joo Lee, Jin Jea Sung, Jong-Won Chung, Woo-Keun Seo, Gyeong-Moon Kim, Tae Keun Jee, Je Young Yeon, Jong-Soo Kim, Jiho Jang

**Affiliations:** 1 S&E bio Co., Ltd., Seoul 06351, Republic of Korea; 2 Translational and Stem Cell Research Laboratory on Stroke, Samsung Medical Center, Seoul 06351, Republic of Korea; 3 Department of Neurology, Samsung Medical Center, Sungkyunkwan University School of Medicine, Sungkyunkwan University, 81 Irwon-ro, Gangnam-gu, Seoul 06351, Republic of Korea; 4 Department of Physiology, Yonsei University College of Medicine, Seoul 03722, Republic of Korea; 5 Department of Neurosurgery, Samsung Medical Center, Sungkyunkwan University School of Medicine, Seoul 06351, Republic of Korea; 6 Department of Biomedical Sciences, College of Medical Convergence, Catholic Kwandong University, Gangneung-si 25601, Republic of Korea

**Keywords:** moyamoya disease, extracellular vesicles, EV-miRNA, HMGB2, endothelial dysfunction, angiogenesis

## Abstract

**Highlights:**

**What are the main findings?**

- Circulating EV-miR-558 was consistently elevated in adult moyamoya disease and showed moderate diagnostic discrimination from intracranial atherosclerosis and healthy controls.
- miR-558 directly targeted HMGB2 and impaired angiogenic function in HUVECs, while patient-derived iPSC-endothelial cells showed concordant miR-558/HMGB2 dysregulation and impaired tube formation.

**What are the implications of the main findings?**

- Circulating EV-miR-558 is a promising candidate biomarker associated with endothelial dysfunction in adult moyamoya disease, although its clinical utility requires further validation.
- The miR-558/HMGB2 axis provides a potential link between circulating EV-miRNA dysregulation and endothelial dysfunction, although EV-mediated transfer remains to be established.

**Abstract:**

Moyamoya disease (MMD) is a progressive cerebrovascular disorder characterized by intracranial arterial stenosis and abnormal collateral vessel formation. The molecular mechanisms by which extracellular vesicle (EV)-derived microRNAs contribute to endothelial dysfunction remain poorly understood. We investigated whether circulating extracellular vesicle-derived microRNAs (EV-miRNAs) contribute to endothelial dysfunction and serve as functional mediators of MMD pathogenesis. Plasma EV-miRNA profiles were compared among patients with MMD, intracranial atherosclerosis (ICAS), and healthy controls, and differentially expressed EV-miRNAs were validated in an independent cohort. Functional studies were performed in human umbilical vein endothelial cells and patient-derived induced pluripotent stem cell-derived endothelial cells. Three EV-miRNAs were significantly upregulated in MMD, among which miR-558 showed the strongest diagnostic performance. miR-558 overexpression impaired endothelial tube formation and proliferation, whereas its inhibition enhanced angiogenic activity. Mechanistically, HMGB2 was identified as a direct target of miR-558, and miR-558 overexpression reduced HMGB2 protein expression. Patient-derived endothelial cells recapitulated increased miR-558 expression, reduced HMGB2 levels, and impaired angiogenic capacity. These findings identify circulating EV-miR-558 a potential biomarker and show that miR-558 suppresses HMGB2 and impairs endothelial angiogenesis in adult MMD, suggesting that the EV-miR-558/HMGB2 pathway represents a potential mechanism underlying endothelial dysfunction and therapeutic target for MMD.

## 1. Introduction

Moyamoya disease (MMD) is a progressive cerebrovascular disorder characterized by chronic stenosis of the terminal internal carotid arteries and the development of abnormal basal collateral vessels known as moyamoya vessels [1]. Although RNF213 is the major genetic susceptibility factor for MMD, the molecular mechanisms linking genetic susceptibility to endothelial dysfunction and aberrant vascular remodeling remain poorly understood [1,2,3]. Not all patients with MMD carry RNF213 variants, and the distribution of pathogenic variants differs substantially among ethnic populations, suggesting that additional molecular regulators and environmental factors contribute to disease development [1,4,5]. Furthermore, the observation that only a small proportion of individuals carrying RNF213 variants develop clinical MMD indicates that genetic susceptibility alone is insufficient to explain disease onset or progression [5].

Furthermore, reliable circulating biomarkers that distinguish MMD from intracranial atherosclerosis (ICAS), a common differential diagnosis in Asian populations, are still lacking [6,7]. Accurate differentiation between MMD and ICAS is clinically important because their therapeutic strategies differ substantially. Medical therapy and endovascular intervention are effective for ICAS, whereas MMD primarily requires surgical revascularization, and ICAS-directed treatments may be ineffective or even harmful in patients with MMD [8,9,10]. Conventional angiography remains the diagnostic gold standard; however, it is invasive and associated with procedural risks. Moreover, the characteristic basal collateral vessels are often less prominent in adult-onset MMD than in childhood-onset disease, making diagnosis more challenging [11]. Consequently, increasing efforts have focused on developing non-invasive diagnostic approaches, including high-resolution magnetic resonance imaging and circulating biomarkers such as endothelial progenitor cells, cytokines, proteins, and genetic factors [2,3,6,7].

MicroRNAs (miRNAs) are small non-coding RNAs that regulate post-transcriptional gene expression by binding to target messenger RNAs and play critical roles in diverse physiological and pathological processes [12]. Extracellular vesicles (EVs), including small EVs, are membrane-bound nanoparticles that mediate intercellular communication by transporting biologically active cargo, including miRNAs and proteins, thereby regulating recipient cell function under physiological and pathological conditions [13]. Circulating EV-derived miRNAs are remarkably stable in the bloodstream and have emerged as promising biomarkers and functional mediators in cardiovascular and cerebrovascular diseases [12]. Recent studies have demonstrated that circulating EV-miRNA signatures can discriminate ischemic stroke subtypes and provide mechanistic insights into vascular pathology [14,15].

Based on these observations, we hypothesized that circulating EV-miRNAs contribute to endothelial dysfunction and function as regulators of MMD pathogenesis. To test this hypothesis, we compared plasma EV-miRNA profiles among patients with MMD, ICAS, and healthy controls, validated candidate EV-miRNAs and investigated their mechanistic roles using endothelial cells, patient-derived induced pluripotent stem cell-derived endothelial cells, and target gene analyses.

## 2. Materials and Methods

### 2.1. Patient Selection

**Figure 1:**
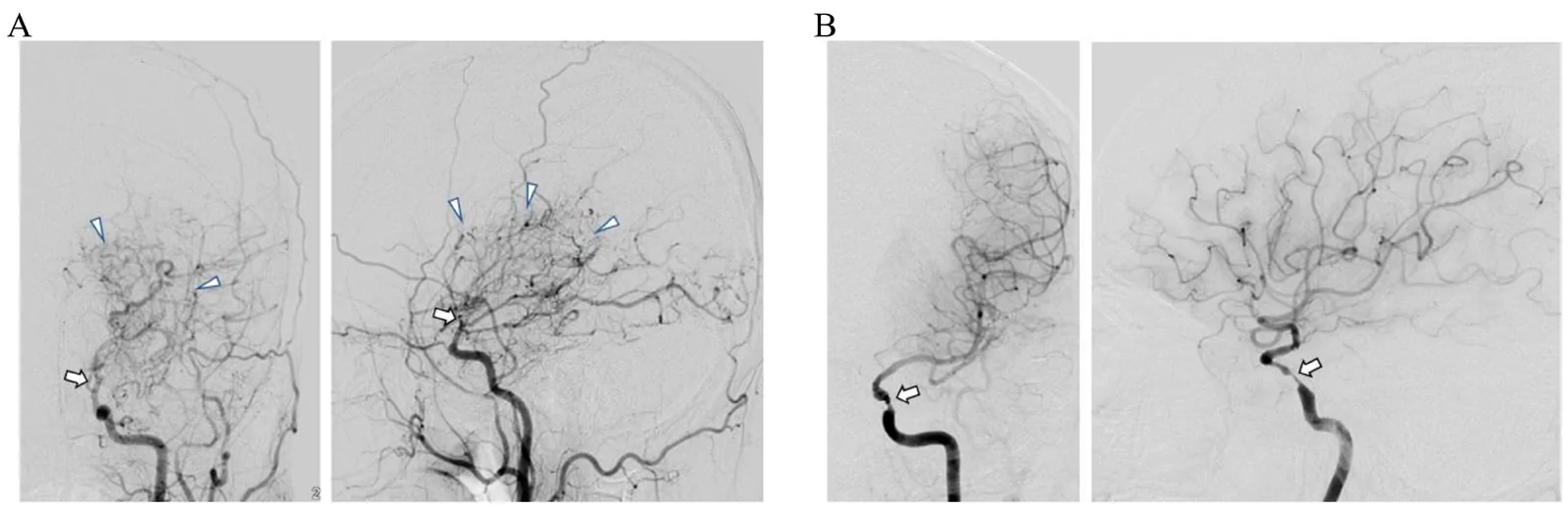
Angiographic features distinguishing moyamoya disease from intracranial atherosclerosis. (**A**) A 47-year-old female patient with moyamoya disease showing distal internal carotid artery (ICA) occlusion (arrow) and moyamoya vessels (arrowhead). (**B**) A 65-year-old male patient with atherosclerotic stenosis on distal ICA (arrow).

Consecutive patients diagnosed with MMD or ICAS and healthy controls were enrolled between January 2014 and January 2017 at Samsung Medical Center. MMD was diagnosed based on stenosis of the distal (terminal) internal carotid artery (ICA) or middle cerebral artery (MCA), accompanied by prominent basal collateral networks on conventional angiography (Figure 1). ICAS was defined as ≥50% stenosis or occlusion of the distal ICA or MCA on magnetic resonance angiography in patients with symptomatic focal or lateralizing neurological deficits within the MCA territory. Patients with extracranial arterial stenosis ≥ 50%, cardio-aortic embolic sources, or moderate-to-severe renal disease were excluded. Age-matched individuals with no history of stroke or MMD, including spouses of patients and healthy volunteers, were enrolled as healthy controls. Participants were first classified as MMD, ICAS, or healthy controls according to the predefined diagnostic criteria. They were then assigned, based on their enrollment dates, to the pooled discovery analysis, the individual validation in the development cohort, or the independent validation cohort. There was no participant overlap among the three analysis sets.

Clinical data including demographics and vascular risk factors were systematically collected. All participants provided written informed consent. This study was conducted in accordance with the principles embodied in the Declaration of Helsinki. The local institutional review board of Samsung Medical Center approved the study protocol (IRB No. 2008-02-046).

### 2.2. Identification of the Ring Finger Protein 213 (RNF213) Mutation

All enrolled patients with MMD or ICAS underwent genetic testing for the RNF213 variant. RNF213 p.Arg4810Lys (c.14429G>A) is a known susceptibility variant for MMD and is also found, although less frequently, in East Asian patients with ICAS. Approximately 20% of patients with ICAS carry this variant [16,17]. Peripheral blood samples were collected during hospital admission, and genomic DNA was purified from leukocytes using the Wizard Genomic DNA Purification Kit (Promega, Madison, WI, USA), according to the manufacturer’s instructions. The RNF213 p.Arg4810Lys mutation (GenBank accession No. NM_001256071.1) was amplified using custom-designed primers, which are available upon request. PCR amplicons generated using a 9700 thermal cycler (Applied Biosystems, Foster City, CA, USA) were directly sequenced using the BigDye Terminator Cycle Sequencing Kit on an ABI Prism 3730xl Genetic Analyzer (Applied Biosystems, Foster City, CA, USA), as previously described [17].

### 2.3. Isolation of Extracellular Vesicles

Citrate plasma sample was prepared by centrifugation of citrated whole blood at 2000× *g* for 15 min and stored at −80 °C until further processing. For EV isolation, the plasma was clarified by centrifugation at 1000× *g* for 15 min at 4 °C to remove cellular debris. The supernatant was centrifuged at 19,800× *g* for 20 min, after which the resulting supernatant was ultracentrifuged at 100,000× *g* for 1 h to collect the sEV-enriched fraction. The pellets collected at 19,800× *g* and 100,000× *g* were designated as the EV fraction and small EV-enriched fraction, respectively. All centrifugation procedures were performed at 4 °C using an Optima TLX ultracentrifuge equipped with a TLA120.2 rotor (Beckman Coulter, Brea, CA, USA). For washing, the pellets were resuspended in filtered phosphate-buffered saline (PBS) and centrifuged again. The final EV-containing pellets were resuspended in 100 µL of PBS and stored at −80 °C until further analysis.

### 2.4. Characterization of Extracellular Vesicles

Nanoparticle tracking analysis was performed after dilution of the EV preparations in vesicle free water. A NanoSight NS300 system (Malvern Panalytical, Worcestershire, UK) was used to measure EV particle concentration and size distribution. The mean and mode size and concentration (particles/mL) were calculated from three independent measurements.

EV morphology was evaluated by TEM and cryo-TEM. For TEM, EVs were fixed with 1% OsO_4_ in 0.1 M phosphate buffer, adsorbed onto Formvar-coated EM grids, stained with 2% uranyl acetate and examined using a JEM-1011 microscopy (JEOL, Tokyo, Japan). For Cryo-TEM, R1.2/1.3 carbon grids (Quantifoil Micro Tools, Großlöbichau, Germany) were rendered hydrophilic by glow discharge (Pelco easiGlow, Ted Pella, Redding, CA, USA). A 4 µL sample was loaded onto a grid, blotted for 1.5 s at 4 °C and 100% humidity, and plunge-frozen in liquid ethane with a Vitrobot Mark IV (FEI, Eindhoven, The Netherlands). The vitrified samples were imaged at 120 kV using a Talos L120C cryo-electron microscope (FEI, Eindhoven, The Netherlands).

Total EV protein was quantified using a protein microBCA assay (Thermo Fisher Scientific, Waltham, MA, USA). As an index of EV purity, the particle-to-protein ratio was calculated by dividing the particle concentration determined by NTA by the corresponding total protein concentration. For Western blotting, 15 µg of EV protein was subjected to SDS-PAGE, transferred to nitrocellulose membranes, and probed with antibodies against EV-positive markers CD63, CD81, Flotillin-1, and TSG101, and the negative marker calnexin. Signals were visualized using horseradish peroxidase-conjugated secondary antibodies and chemiluminescence detection reagents (Thermo Fisher Scientific, Waltham, MA, USA).

### 2.5. miRNA Profiling and Validation

EV-RNA was isolated with the miRNeasy Serum/Plasma Kit (Qiagen, Hilden, Germany), according to the manufacturer’s instructions. Before isolation, synthetic *Caenorhabditis elegans* miR-39 was spiked into each sample for inter-sample variation. RNA was recovered in 14 µL of RNase-free water and stored at −80 °C. RNA concentration was measured using a NanoDrop 1000 spectrophotometer (Thermo Fisher Scientific, Waltham, MA, USA).

For miRNA profiling, pooled EV-RNA samples (*n* = 10 from each group) were analyzed using the Human Whole-miRNome Array and the miScript SYBR Green PCR Kit (Qiagen, Hilden, Germany). Reverse transcription was performed using 4 µL of RNA and the miScript II RT Kit. qRT-PCR was carried out in 384-well plates using an ABI 7900HT system (Thermo Fisher Scientific, Waltham, MA, USA) with synthetic spike-ins and reference miRNAs for normalization. Normalized Ct values were calculated using global Ct mean normalization. As the initial miRNA profiling was performed using pooled EV-RNA samples, no multiple-testing correction, including FDR adjustment, was applied.

To determine the findings of the array analysis, selected miRNAs were quantified by qPCR in an independent individual cohort (MMD: *n* = 40; ICAS: *n* = 22; HC: *n* = 39) using the 2^−ΔCt^ method. Only miRNAs with Ct < 35 and >5-fold increase compared with HC were considered for further analysis. Candidate miRNAs were further evaluated in an additional independent validation cohort (MMD: *n* = 24; ICAS: *n* = 18). TaqMan^®^ MicroRNA Assays (Applied Biosystems, Foster City, CA, USA) were used for qPCR. All assays were carried out by investigators who were blinded to clinical data.

### 2.6. In Vitro Studies

#### 2.6.1. Cell Culture and miRNA Transfection

Human umbilical vein endothelial cells (HUVECs) were maintained in M199 medium (Gibco, Grand Island, NY, USA) supplemented with 20% fetal bovine serum (FBS), 5 U/mL heparin, and 3 ng/mL basic fibroblast growth factor (bFGF) at 37 °C in 5% CO_2_. For miRNA transfection, 4 × 10^5^ cells were seeded in 60 mm dishes and transfected with 30 nM miRNA mimics or 100 nM inhibitors using Lipofectamine™ RNAiMAX (Thermo Fisher Scientific, Waltham, MA, USA) for 6 h according to the manufacturer’s protocol. Reagents for miRNA assays were purchased from Bioneer (Daejeon, Republic of Korea).

#### 2.6.2. Cell Proliferation Assays

Transfected cells were seeded at 1.7 × 10^4^ cells/well in 6-well plates containing M199 medium supplemented with 20% FBS, 5 U/mL heparin, and 3 ng/mL bFGF. After 3 days of incubation at 37 °C in 5% CO_2_, cells were harvested using trypsinization and manually counted using a hemocytometer.

#### 2.6.3. Cell Migration Assays

HUVECs were cultured for 48 h post-transfection and seeded at 1.7 × 10^4^ cells/well in 6-well plates. After reaching confluence, they were serum starved for 2 h and treated with mitomycin C to inhibit their proliferation. A vertical scratch was made using a sterile pipette tip, and images of the wound area were captured at 0 and 7 h using a phase-contrast microscope. The wound area was measured using ImageJ software 1.53a (National Institutes of Health, Bethesda, MD, USA).

#### 2.6.4. Tube Formation Assays

To assess the pro-angiogenic effects of candidate miRNAs, transfected HUVECs were seeded at 1.7 × 10^4^ cells/well on growth factor-reduced Matrigel (BD Biosciences, San Jose, CA, USA) in µ-Slides Angiogenesis (ibidi, Gräfelfing, Germany). Cells were incubated for 4 h at 37 °C with 5% CO_2_ to allow tube formation. Phase-contrast images were captured using an Olympus microscope, and the number of tube-like structures was quantified using ImageJ software at 4× magnification.

#### 2.6.5. Western Blot Analysis

HUVECs were lysed in the RIPA buffer containing protease inhibitors. Protein lysates (10 µg per sample) were separated using SDS-PAGE (Thermo Fisher Scientific, Waltham, MA, USA) and transferred onto 0.45 µm nitrocellulose membranes (Bio-Rad, Hercules, CA, USA). After blocking, the membranes were incubated overnight at 4 °C with primary antibodies against MTA2 (ab8106, Abcam, Cambridge, UK), CAV1 (3238S, Cell Signaling Technology, Danvers, MA, USA), HMGB2 (ab67282, Abcam, Cambridge, UK), and GAPDH (2118S, Cell Signaling Technology, Danvers, MA, USA). Following washes with Tris-buffered saline with Tween 20 (TBS-T), horseradish peroxidase-conjugated secondary antibodies were applied for 1 h at room temperature. Protein bands were visualized using a Fusion SOLO S chemiluminescence imaging system (Vilber Lourmat, Collégien, France) and quantified using ImageJ software. Target protein expression was normalized to that of GAPDH.

#### 2.6.6. Luciferase miRNA Reporter Assays

HEK293 cells (1.5 × 10^6^) were transfected with miR-558, miR-623 or miR-548b-3p mimic. After 24 h, 8 × 10^4^ cells were seeded into 24-well plates and incubated overnight. The cells were then transfected with 100 ng of the corresponding reporter vector. Following transfection, cells were lysed using 5× Passive Lysis Buffer (Promega, Madison, WI, USA) and luciferase activity was measured using the Dual-Glo^®^ Luciferase Assay System (Promega, Madison, WI, USA), according to the manufacturer’s instructions. Firefly luciferase activity was normalized to Renilla luciferase activity. Luminescence was measured using a Synergy HTX Multimode Reader (BioTek Instruments, Winooski, VT, USA).

### 2.7. Experiments on Patient-Specific Induced Pluripotent Stem Cells (iPSCs)

#### 2.7.1. Generation of Patient-Specific iPSCs

Peripheral blood mononuclear cells (PBMCs) were separated from whole blood obtained from patients and healthy controls using SepMate™ PBMC Isolation Tubes (STEMCELL Technologies, Vancouver, BC, Canada) and Ficoll-Paque PLUS (GE Healthcare, Chicago, IL, USA) according to the manufacturers’ instructions. Reprogramming of PBMCs was performed using the CytoTune^®^-iPS 2.0 Sendai Reprogramming Kit (Thermo Fisher Scientific, Waltham, MA, USA) under standard conditions. After 60 d, transgene-free iPSC colonies showing typical pluripotent stem cell morphology were manually selected and clonally expanded. Pluripotency was confirmed as described previously [18].

#### 2.7.2. Differentiation of iPSC-ECs

Endothelial cell (EC) differentiation of iPSCs was performed using a two-dimensional monolayer differentiation protocol [19]. On day 0, iPSCs were cultured for 2 d in differentiation medium consisting of RPMI and B-27 supplement minus insulin (Thermo Fisher Scientific, Waltham, MA, USA) with 6 mM CHIR-99021 (Axon Medchem, Groningen, The Netherlands). The cells were then maintained for an additional 2 d in differentiation medium with 3 mM CHIR-99021. On day 4, the medium was replaced with a differentiation medium supplemented with 50 ng/mL VEGF (PeproTech, Cranbury, NJ, USA) and 10 ng/mL bFGF (PeproTech, Cranbury, NJ, USA) for 5 d. On day 9, iPSC-ECs were sorted for CD144+ using MACS and cultured in EGM-2 medium (Lonza, Basel, Switzerland) on gelatin-coated plates. The endothelial phenotype was verified by staining for CD31, CD144, and CD45 followed by FACS analysis.

#### 2.7.3. Tube Formation of iPSC-ECs

Growth factor-reduced Matrigel (356231, BD Biosciences, San Jose, CA, USA) was used to coat μ-Slides Angiogenesis (81506, ibidi, Gräfelfing, Germany). iPSC-ECs were seeded at a density of 1 × 10^4^ cells per well onto pre-coated μ-slides and incubated for 4 h at 37 °C in a CO_2_ incubator. The tube-like structures were imaged using phase-contrast microscopy. Angiogenic activity was quantified by measuring the number of loops with the Angiogenesis Analyzer plugin in the ImageJ software.

#### 2.7.4. Bioinformatic Analysis of miRNAs

After confirming data normalization (miRNA profiles were analyzed using the Human Whole-miRNome Array), differentially expressed miRNAs showing a >2-fold difference in average signal values among the HC, MMD, and ICAS groups were manually selected. Hierarchical clustering analysis was performed using Multi Experiment Viewer (MEV) software version 4.9.0 to identify groups of miRNAs with similar expression patterns. Gene ontology (GO) and Kyoto Encyclopedia of Genes and Genomes (KEGG) pathway enrichment analyses of the predicted target genes were conducted using the miRWalk 2.0 web-based tool (http://mirwalk.umm.uni-heidelberg.de; accessed on 10 August 2018). Potential miRNA targets were predicted using the RNA22 database (https://cm.jefferson.edu/rna22/; accessed on 10 August 2018).

#### 2.7.5. qPCR Verification of miRNAs and mRNAs in iPSC-ECs

To quantify the expression of the three selected miRNAs (miR-623, miR-558, miR-548b-3p) and RNF213 in patient-specific iPSC-ECs, total RNA was extracted using TRIzol (15596026, Thermo Fisher Scientific, Waltham, MA, USA) according to the manufacturer’s instructions. RNA concentrations were measured using a NanoDrop 1000 spectrophotometer. miRNA expression was confirmed using TaqMan^®^ MicroRNA Assays (Applied Biosystems, Foster City, CA, USA) and analyzed using the 2^−ΔΔCt^ quantification method. cDNA was synthesized from 1 µg of total RNA using Superscript III reverse transcriptase (Thermo Fisher Scientific, Waltham, MA, USA) according to the manufacturer’s instructions. mRNA expression was determined by qPCR using SYBR Green PCR master mix (Applied Biosystems, Foster City, CA, USA) on a QuantStudio 6 Real-Time PCR System (Applied Biosystems, Foster City, CA, USA). Primer sequences for CAV1, MTA2, HMGB2, RNF213, and GAPDH were as follows. CAV1 (Forward 5′-TCATCTGGGGCATTTACTTCG-3′, Reverse 5′-CAGCTTCAAAGAGTGGGTCAC-3′), MTA2 (Forward 5′-CATGAATGGGGCTGGATTTCA-3′, Reverse 5′-CAGTCCCCCATACTTCTTCCA-3′), HMGB2 (Forward 5′-CTCGCTATGACAGGGAGATGA-3′, Reverse 5′-GCCAGGGTGTTCACTTTTGAT-3′), RNF213 (Forward 5′-CCTGAGTCAAAAGGAGGCAG-3′, Reverse 5′-GCTCAGCTACAGCATCAACT-3′). Relative gene expression was calculated using the 2^−ΔΔCt^ method with GAPDH as an internal control.

### 2.8. Statistical Analyses

Differences in discrete variables between the groups were evaluated using the χ^2^ or Fisher’s exact test. Normality of continuous clinical variables was assessed using the Shapiro–Wilk test. Differences in continuous variables were evaluated using ANOVA, Kruskal–Wallis test, *t*-test or Mann–Whitney *U* tests. Post hoc comparisons between the three groups were performed, and Dunnett’s method was used to correct for multiple tests. Receiver operating characteristic (ROC) curves were used to compare the discrimination power of each plasma EV-miRNA and the combination of miRNAs for predicting MMD. Discrimination power was assessed by calculating the area under the ROC curve (AUC). An area of 1 implies that the test has perfect sensitivity and specificity, whereas an area of 0.5 implies that the model’s predictions lack predictive accuracy. The best model was defined as the one with the largest AUC. All group comparisons and ROC analyses were unadjusted. A two-tailed *p* value of <0.05 was considered significant. All statistical analyses were performed using commercially available software (IBM SPSS Statistics version 24.0, IBM Corp., Armonk, NY, USA).

## 3. Results

### 3.1. Patient Characteristics

A total of 173 patients were recruited for this study, of whom 74 had MMD, 50 had ICAS, and 49 were healthy controls (HCs). Patient characteristics in the development and validation cohorts are summarized in Table 1.

Compared with the MMD and HC groups, patients in the ICAS group were older, more often male, and had a significantly higher prevalence of vascular risk factors (*p* < 0.01 for all). ICAS patients also had significantly higher statin use than MMD patients (e.g., statin use: 98.0% vs. 32.4%). In terms of clinical presentation, ischemic stroke was significantly more frequent in ICAS (84.0%) than in MMD (47.3%, *p* < 0.001), whereas intracerebral hemorrhage occurred only in the MMD group (12.2%), consistent with the known hemorrhagic tendency of moyamoya vasculopathy. Between the MMD and HC groups, HC participants were significantly older than MMD patients (*p* = 0.011), but sex distribution and vascular risk factor prevalence did not differ significantly (*p* > 0.05).

### 3.2. Circulating EV-miRNA Profiling Identifies miR-558 as a Candidate Biomarker for MMD

**Figure 2:**
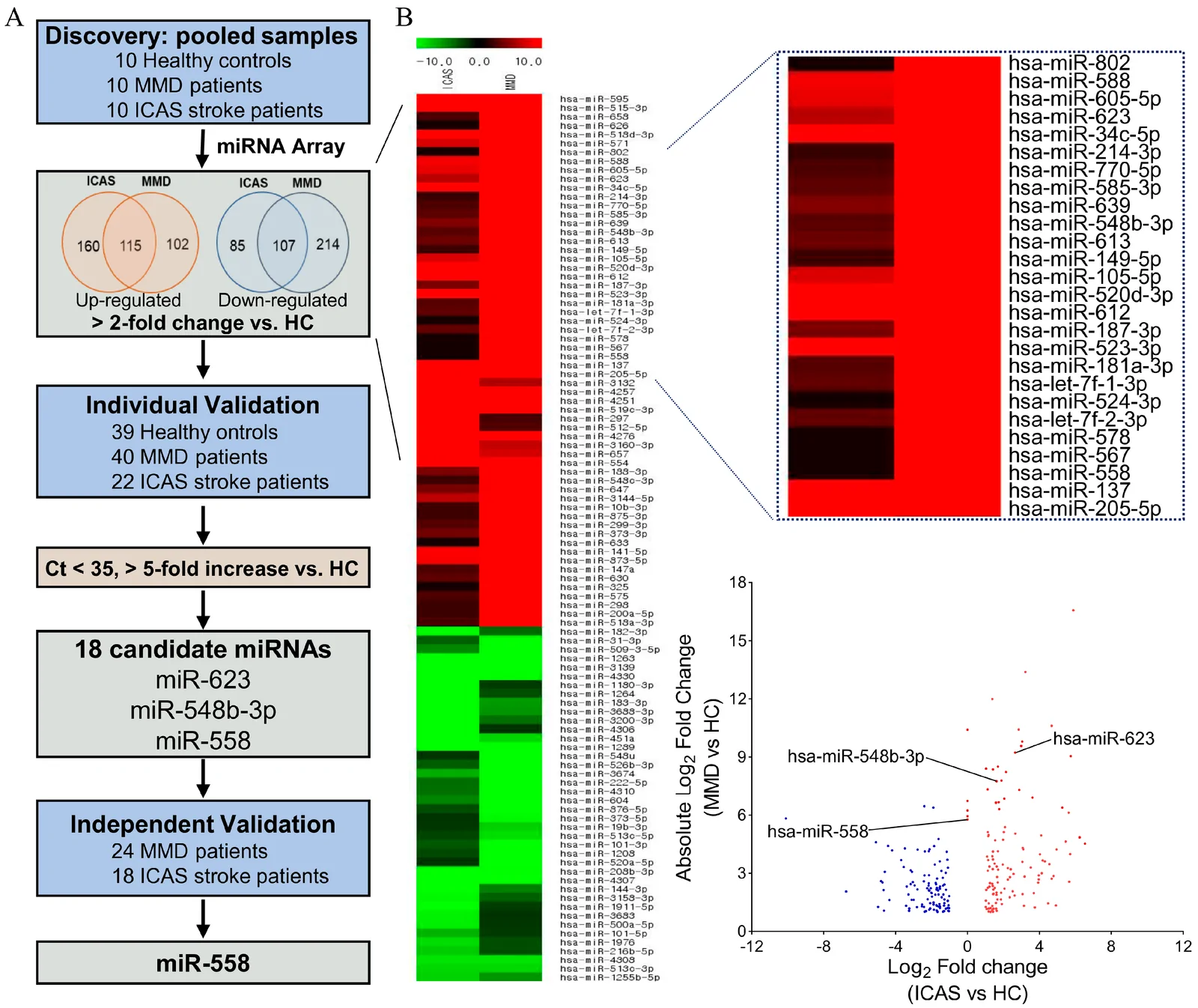
Plasma extracellular vesicle miRNA profiling identifies MMDassociated EV-miRNAs. (**A**) Overview of discovery and validation flow. Pooled miRNA profiling was followed by individual qPCR analysis. Eighteen miRNAs met the criteria of Ct < 35 and a >5-fold increase compared with healthy controls (HCs), from which three candidates were selected for independent validation. The Venn diagrams show miRNAs with >2-fold increases or decreases relative to HCs. (**B**) Heatmap and two-dimensional scatter plot of discovery miRNAs. Hierarchical clustering of the miRNA profiles compared both HCs vs. ICAS and HCs vs. MMD. Color scale indicates Log2 normalized fold changes. A two-dimensional plot shows miRNAs that were increased (red) or decreased (blue) in both ICAS vs. HCs and MMD vs. HCs. The X-axis represents Log2-fold changes in ICAS vs. HCs and Y-axis represents the absolute value of Log2-fold changes in MMD vs. HCs.

EVs were isolated from plasma of healthy controls, patients with moyamoya disease, and ICAS patients using ultracentrifugation. EV characterization, including particle size, morphology, particle-to-protein ratios, and EV marker analysis, is presented in Figure S1. Plasma EV-derived miRNA profiling revealed distinct expression patterns between patients with MMD, ICAS, and HCs (*n* = 10 per group). Compared to HCs, 275 miRNAs were upregulated in patients with ICAS and 217 in patients with MMD, with 115 miRNAs overlapping between the two groups. Among miRNAs showing a >5-fold increase (Ct < 35), 11 were shared by both disease groups, while 7 were uniquely elevated in patients with MMD (Figure 2A).

Hierarchical clustering analysis demonstrated distinct group-specific miRNA expression profiles. Volcano plots further illustrated significantly upregulated and downregulated miRNAs in both the ICAS and MMD groups compared to HCs (Figure 2B). Notably, several miRNAs were consistently upregulated in patients with MMD, suggesting their potential as disease-specific biomarkers.

**Figure 3:**
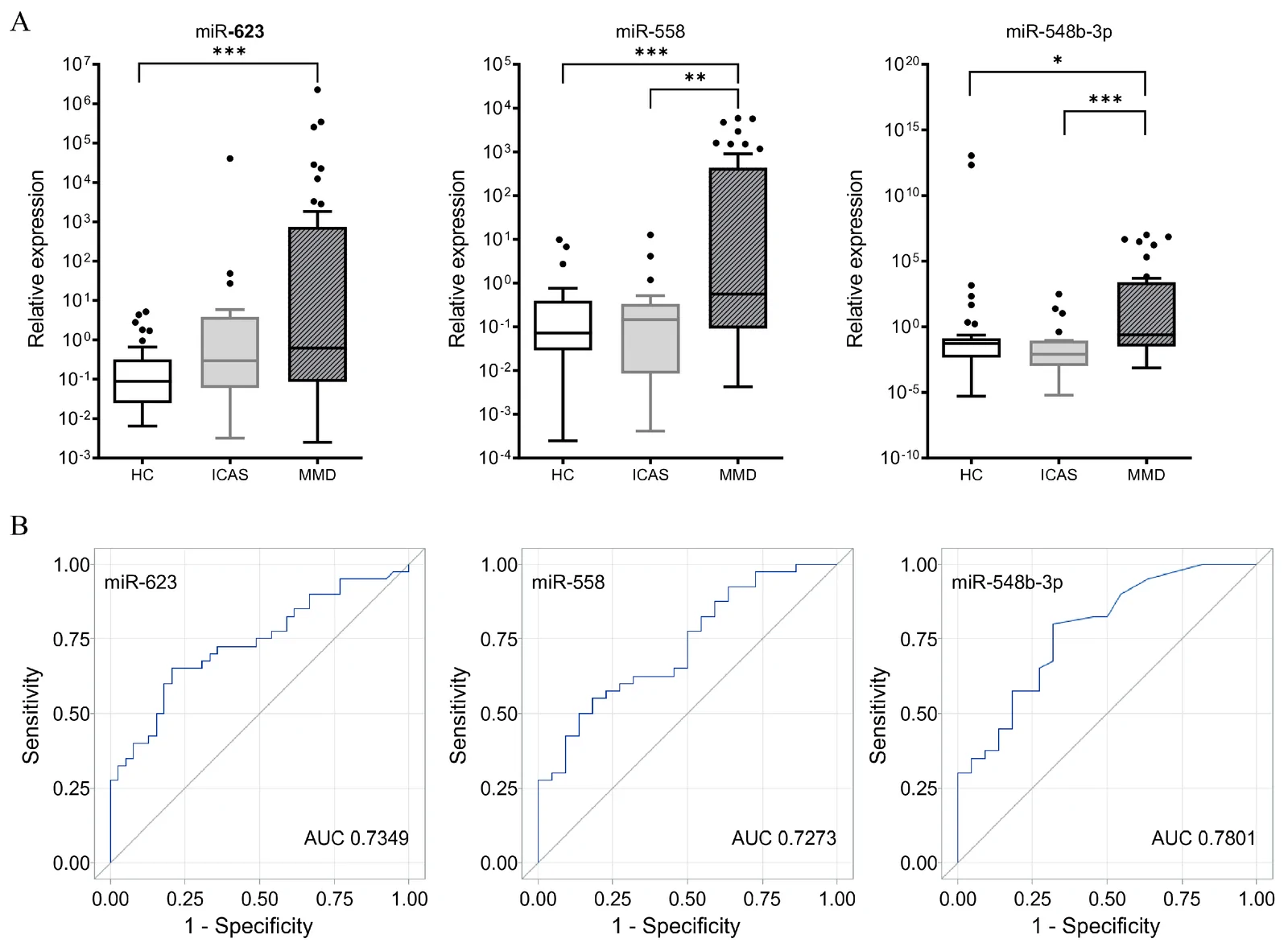
Candidate plasma EV-miRNAs distinguish MMD from ICAS and healthy controls. (**A**) qPCR results of relative abundance of EV-miRNAs in patients with MMD compared with those with ICAS and HC. (**B**) Receiver operating characteristic (ROC) curve for MMD in patients with intracranial stenosis. * *p* < 0.05. ** *p* < 0.01. *** *p* < 0.001.

Based on the individual qPCR results, three candidate miRNAs—miR-623, miR-558, and miR-548b-3p—were selected for further validation. All three were significantly upregulated in individual plasma EV samples from patients with MMD compared to HCs (*p* < 0.05). Specifically, miR-558 and miR-548b-3p levels were significantly higher in patients with MMD compared to those with ICAS (*p* = 0.0033 and *p* = 0.0004, respectively) (Figure 3A).

ROC curve analysis supported their diagnostic utility, with AUCs of 0.7349 (miR-623), 0.7273 (miR-558), and 0.7801 (miR-548b-3p) (Figure 3B). These results suggest that miR-548b-3p and miR-558 may serve as promising plasma EV-derived biomarkers for distinguishing patients with MMD, both from those with ICAS and healthy individuals.

### 3.3. Independent Validation Identifies Circulating EV-miR-558 as the Most Robust Biomarker

**Figure 4:**
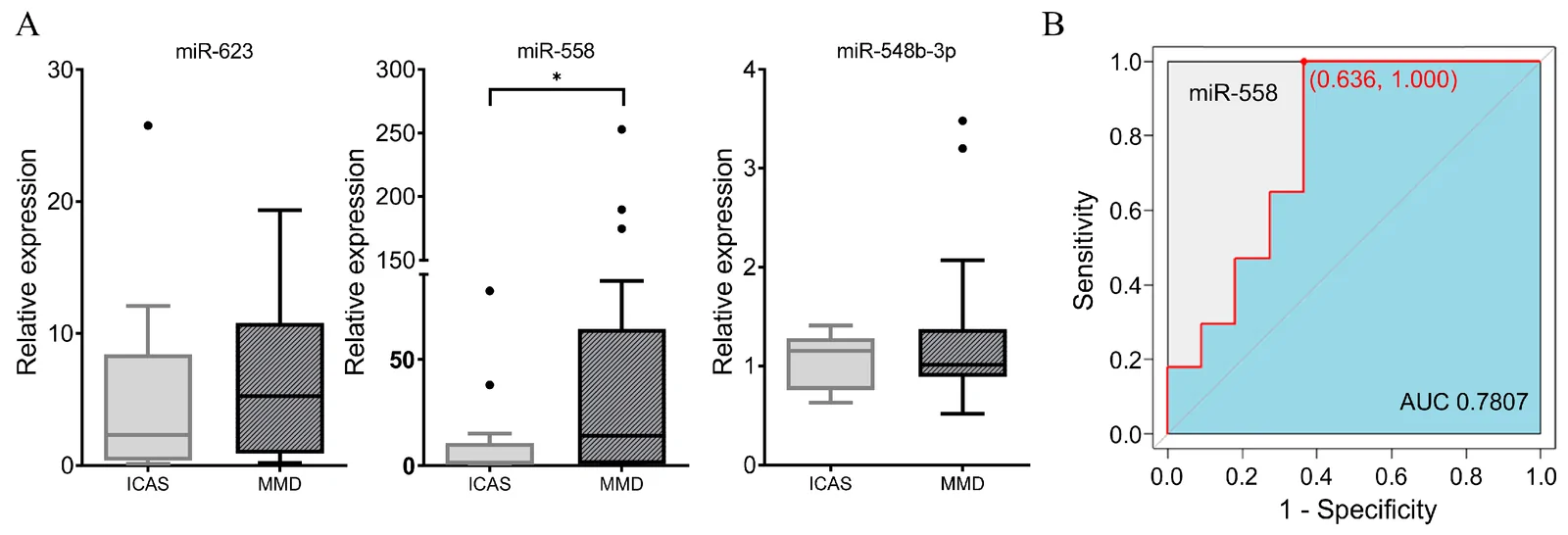
Independent validation identifies EV-miR-558 as the most robust MMD-associated EV-miRNA. (**A**) qPCR results of the relative abundances of EV-miR-623, miR-558, and miR-548b-3p in patients with MMD compared with those with ICAS. (**B**) Receiver operating characteristic (ROC) curve for MMD in patients with intracranial stenosis. The cutoff score was 0.72-fold for miR-558 (as normalized with miR-39, a spike-in control). * *p* < 0.05.

To validate the diagnostic potential of the candidate miRNAs, their expression levels were assessed using qRT-PCR in an independent cohort of 24 patients with MMD and 18 patients with ICAS. Among the three candidates, miR-558 remained significantly elevated in patients with MMD compared to patients with ICAS, whereas expression levels of miR-623 and miR-548b-3p were not significantly different between the MMD and ICAS groups (Figure 4A).

ROC analysis demonstrated that plasma EV-derived miR-558 had moderate discriminative power for identifying MMD, with an AUC of 0.7807 (95% CI: 0.5778–0.9837) and an optimal cutoff value of 0.72 (Figure 4B). At this threshold, miR-558 showed a sensitivity of 100%, a specificity of 63.6%, and a diagnostic accuracy of 81.8%, supporting its clinical potential as a promising candidate biomarker for MMD.

### 3.4. miR-558 Impairs Endothelial Function and Directly Targets HMGB2

#### 3.4.1. Bioinformatic Analysis Identifies HMGB2 as a Candidate Target of miR-558

**Figure 5:**
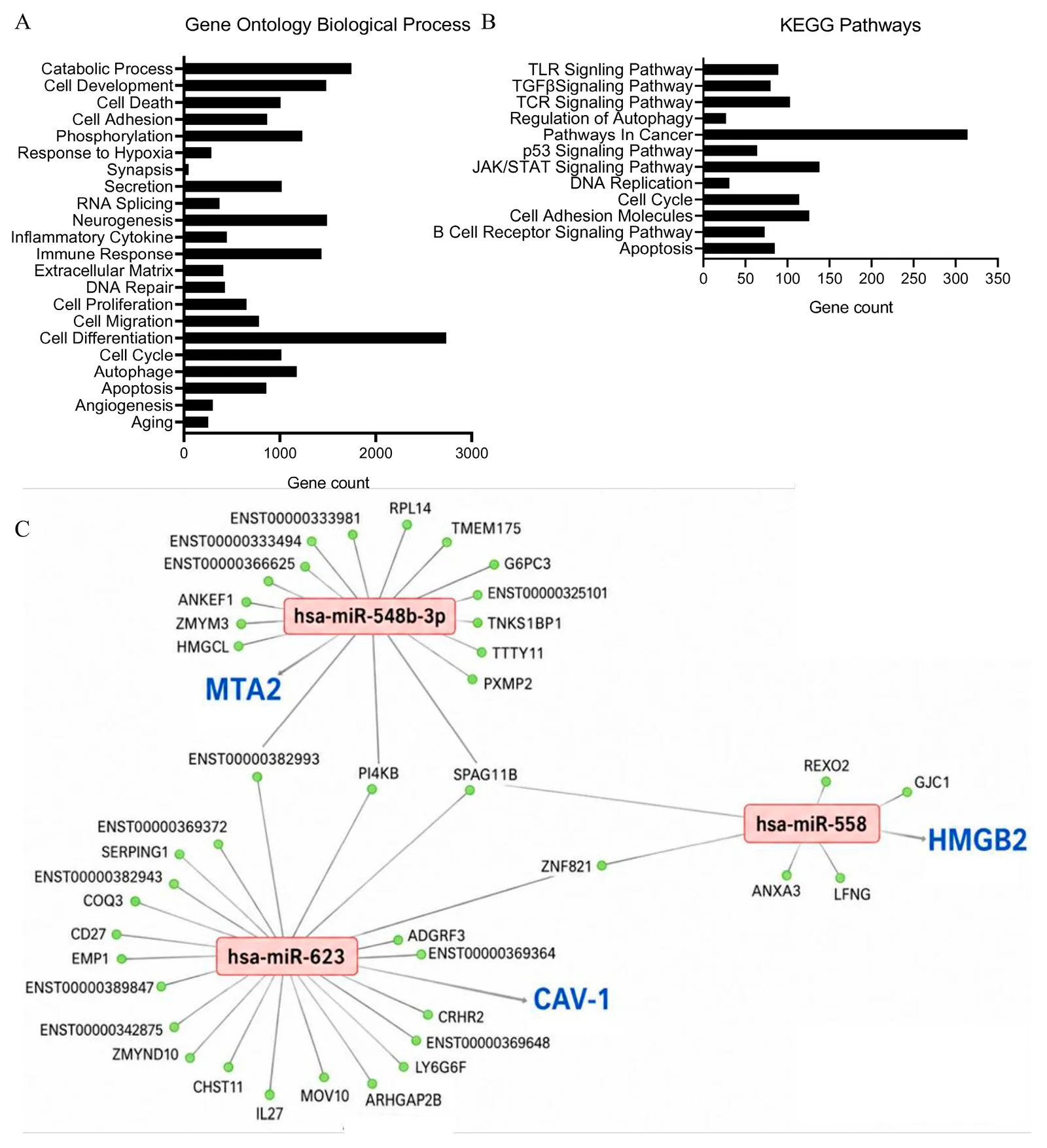
Bioinformatic analyses identify endothelial and inflammatory pathways targeted by MMD-associated EV-miRNAs. (**A**) Gene Ontology (GO) of biological processes. (**B**) Kyoto Encyclopedia of Genes and Genomes (KEGG) pathways. Both GO and KEGG data are presented using only the 10 most relevant terms on target genes of miR-623, miR-558 and miR-548b-3p in extracellular vesicles (EVs). (**C**) miRNA–target gene interaction network.

To explore the pathological relevance of the three miRNAs upregulated in plasma EVs from patients with MMD, a comprehensive bioinformatics analysis was performed using miRWalk-predicted target genes. GO enrichment revealed significant associations with biological processes, such as cell differentiation, migration, proliferation, immune response, development, and angiogenesis (Figure 5A). KEGG pathway analysis further indicated enrichment in signaling pathways critical to endothelial function, including JAK-STAT, TGF-β, T-cell receptor, and Toll-like receptor pathways (Figure 5B).

Network analysis identified multiple putative target genes regulated by miR-548b-3p, miR-558, and miR-623 (Figure 5C). Representative targets included MTA2 (Metastasis Associated 1 Family Member 2), HMGB2 (High Mobility Group Box 2), and CAV1, which are implicated in chromatin remodeling, vascular injury and inflammation, and endothelial structure and remodeling, respectively. These results suggest that dysregulated EV-miRNAs may collectively influence endothelial function and vascular homeostasis in MMD.

Because HMGB2 is a key regulator of vascular injury, endothelial repair, and angiogenesis, and miR-558 was the only candidate consistently validated in an independent cohort, we focused subsequent analyses on the miR-558/HMGB2 interaction.

#### 3.4.2. miR-558 Suppresses Endothelial Angiogenesis and Directly Targets HMGB2

**Figure 6:**
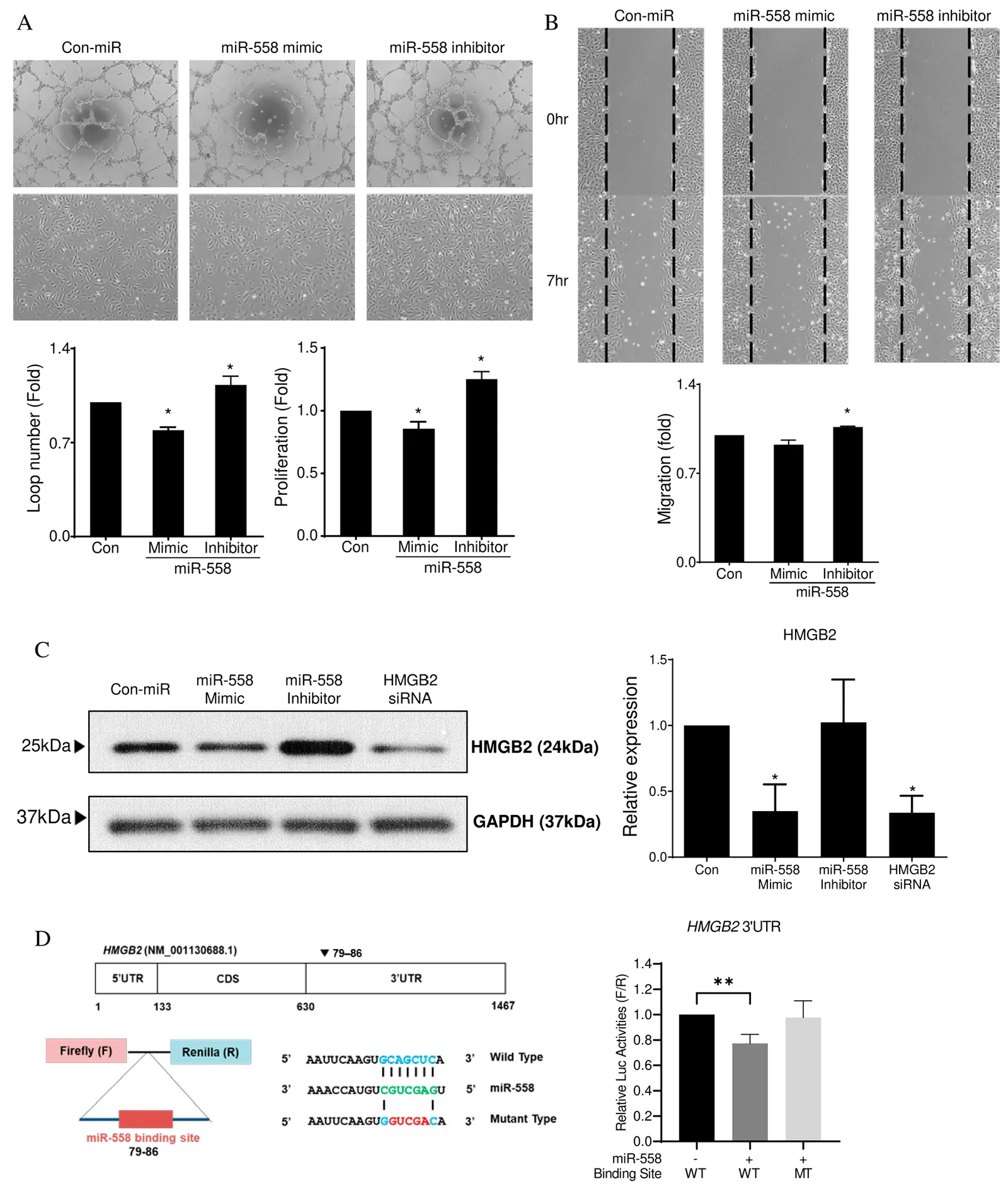
miR-558 suppresses endothelial angiogenic function and targets HMGB2. (**A**–**C**) HUVECs were transfected with control miRNA, miR-558 mimic, or miR-558 inhibitor for tube formation, cell proliferation, and cell migration assays. (**A**) Representative images of tube formation (**upper panels**) and proliferation assays (**lower panels**), with accompanying bar graphs showing quantitative analysis. (**B**) Representative images of migration assays, with accompanying bar graphs showing quantitative analysis. (**C**) Western blotting was performed on HUVEC lysates using antibodies against HMGB2 and GAPDH, and band intensities were quantified by densitometric analysis and normalized to GAPDH. The Western blot images shown are cropped, and the original uncropped blots are no longer available. (**D**) Schematic of HMGB2 transcript and reporter construction (**upper panel**). Luciferase activity in HEK293 cells co-transfected with a Wild-Type (WT) or Mutant (MT) reporter and miR-558 mimic, normalized to Renilla luciferase activity. Data are presented as mean ± SD from three independent experiments (*n* = 3). * *p* < 0.05. ** *p* < 0.01.

To assess the functional roles of miR-558, HUVECs were transfected with miR-558 mimics or inhibitors, followed by tube formation, proliferation, and migration assays. The miR-558 mimic significantly impaired tube formation and reduced cell proliferation, whereas its inhibitor enhanced both these processes (Figure 6A). In the migration assays, miR-558 inhibition significantly promoted cell migration, whereas the mimic had no significant effect (Figure 6B). Western blot analysis confirmed that the miR-558 mimic reduced HMGB2 protein expression (Figure 6C), and luciferase reporter assays confirmed that HMGB2 is a direct target of miR-558 (Figure 6D).

Similarly, transfection with the miR-623 and miR-548b-3p mimics suppressed endothelial tube formation and proliferation (Figures S2 and S3). Both miRNAs reduced the expression of their predicted targets, CAV1 and MTA2. However, only CAV1 was confirmed as a direct target through luciferase reporter analysis.

Collectively, these findings demonstrate that HMGB2 is an experimentally validated direct target of miR-558 and support the miR-558/HMGB2 pathway as a potential link between circulating EV dysregulation and endothelial dysfunction in MMD.

#### 3.4.3. Patient-Derived Endothelial Cells Recapitulate miR-558 Dysregulation and Angiogenic Defects

PBMCs from six patients with MMD and three HCs were successfully reprogrammed into iPSCs, all of which maintained a normal karyotype and expressed the pluripotency markers OCT4 and SSEA4 (Figure S4). These iPSCs were differentiated into endothelial cells (iPSC-ECs), as confirmed by the high expression of CD31 and CD144, along with a lack of CD45 expression. Notably, iPSC-ECs from patients with MMD exhibited reduced expression of endothelial markers and impaired differentiation efficiency compared to HCs (Figure S5).

**Figure 7:**
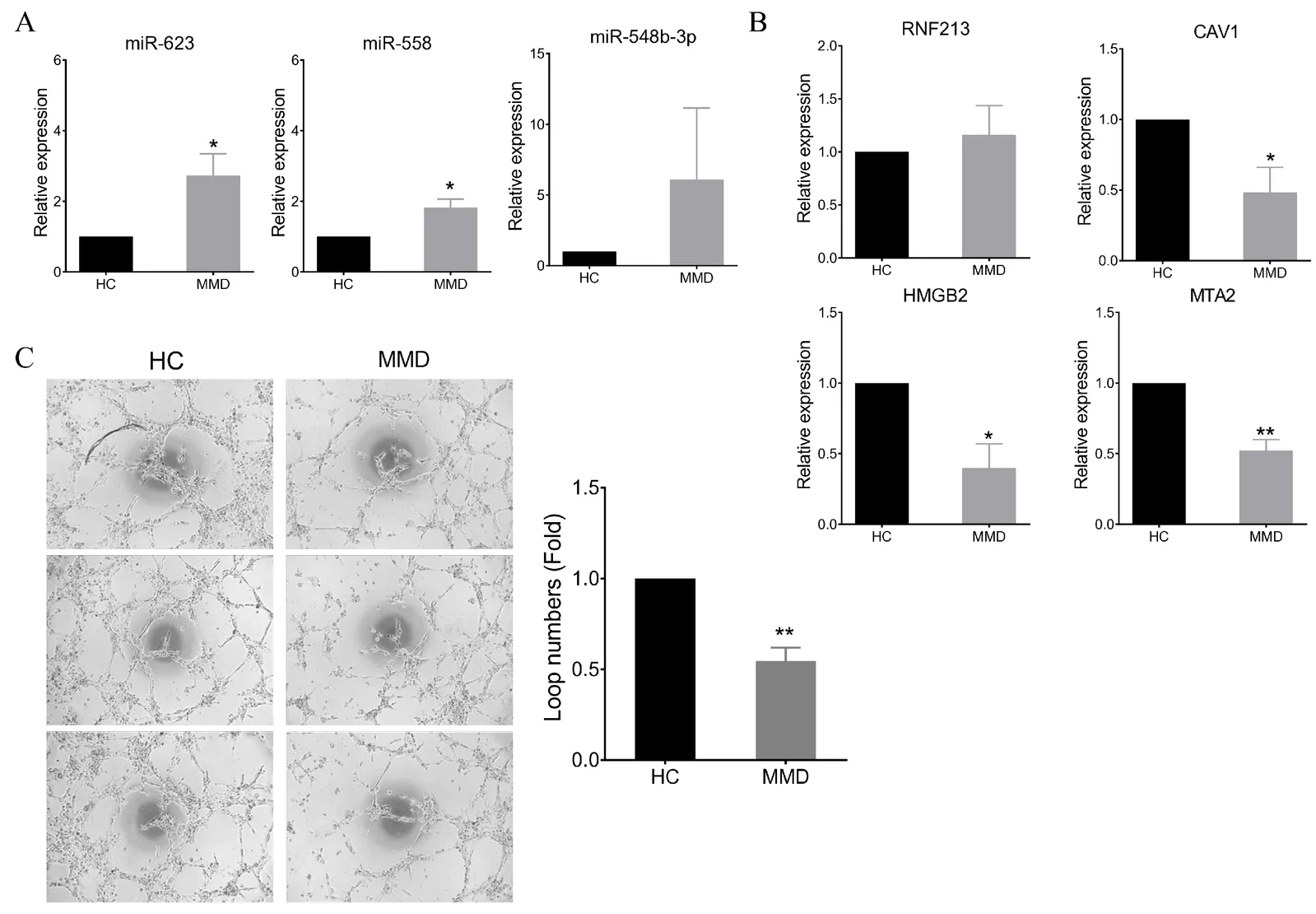
Patient-derived iPSC-endothelial cells recapitulate miR-558/HMGB2 dysregulation and impaired angiogenesis. (**A**) Relative miRNA expression of miR-623, miR-558, miR-548b-3p in iPSC-ECs generated from three healthy controls and three patients with MMD. (**B**) mRNA levels of miRNA target genes and *RNF213* in iPSC-ECs. (**C**) Results of tube formation assays. Representative images of tube formation (**left panel**) and quantitative analysis of loop number by ImageJ using the Angiogenesis Analyzer plugin. Statistical analyses were based on three independent donor-derived iPSC-EC lines per group. Data are presented as mean ± SD. * *p* < 0.05. ** *p* < 0.01.

qPCR analysis showed a significant upregulation of miR-558 and miR-623 in MMD-derived iPSC-ECs, with a non-significant trend for miR-548b-3p (Figure 7A). Correspondingly, the expression of their predicted target genes, *HMGB2*, *CAV1*, and *MTA2*, was significantly downregulated (Figure 7B). In contrast, RNF213 expression did not differ between the iPSC-ECs of MMD and HCs.

Consistent with these molecular changes, MMD-derived iPSC-ECs showed significantly impaired tube formation (Figure 7C), suggesting that angiogenic dysfunction may be driven by dysregulation of the miRNA–target gene network rather than by changes in RNF213 expression. These findings are consistent with a possible role of the miR-558–HMGB2 axis in MMD-related endothelial dysfunction.

## 4. Discussion

In this study, we identified circulating EV-miR-558 as a potential biomarker and mechanistic associated with endothelial dysfunction in adult MMD. Circulating EV-miRNA profiling demonstrated diagnostic potential for differentiating MMD from ICAS in patients with intracranial large artery occlusive disease. By integrating plasma EV profiling, independent cohort validation, patient-derived iPSC-endothelial cells, and functional target analyses, we further demonstrate that miR-558 suppresses HMGB2 and impairs endothelial angiogenic function. Together, these findings support a potential mechanistic framework linking circulating EV-miRNA dysregulation to endothelial dysfunction in MMD and expand current understanding of disease pathogenesis beyond genetic susceptibility.

Our findings support the concept that post-transcriptional regulation by EV-derived miRNAs may represent an additional regulatory mechanism. Several genes involved in vascular remodeling and angiogenesis, including RNF213, CAV1, VEGFA, HIF1α, filamin-A, and NFAT1, are regulated by miRNAs, highlighting the potential contribution of miRNA-mediated regulatory networks to MMD pathogenesis. EV-derived miRNAs function as dynamic signaling molecules that mediate intercellular communication within the vascular microenvironment and respond to pathological and environmental stimuli, in contrast to inherited genetic susceptibility, which remains relatively constant throughout life [13,20]. In this context, the consistent upregulation of miR-558 in both circulating EVs and patient-derived endothelial cells, together with its direct regulation of HMGB2, suggests that EV-miR-558 may represent a molecular interface through which genetic susceptibility is translated into endothelial dysfunction and impaired angiogenic responses in MMD.

The clinical relevance of circulating growth factors and cytokines in MMD remains controversial, as their altered levels may represent secondary responses to chronic ischemia, inflammation, or compensatory collateral formation rather than disease-specific mechanisms [7]. Likewise, most previous studies compared circulating miRNA profiles between patients with MMD and healthy controls without investigating the functional significance of dysregulated miRNAs beyond pathway prediction analyses [21,22,23,24,25].

In this study, we identified three dysregulated EV-miRNAs as potential contributors to MMD pathogenesis. Among them, miR-558 emerged as the principal candidate for mechanistic investigation because it was consistently upregulated in both circulating EVs and patient-derived iPSC-endothelial cells. Bioinformatic prediction followed by functional validation identified HMGB2, a key regulator of vascular injury and repair, as a direct target of miR-558. Functionally, miR-558 overexpression led to HMGB2 downregulation and impaired endothelial angiogenesis in vitro. Consistent with our findings, Uchino et al. also reported dysregulated miRNA expression in patient-derived iPSC-endothelial cells from patients with MMD, supporting the utility of circulating miRNA profiles as indicators of endothelial alterations. Previous studies have shown that EVs can deliver miRNAs to endothelial cells and modulate their function [26]. Collectively, these findings support miR-558 as a functionally relevant regulator of endothelial function and identify the EV-miR-558/HMGB2 pathway as a potential mechanism contributing to endothelial dysfunction in MMD. HMGB2 is a nuclear chromatin-associated protein involved in transcriptional regulation and has been implicated in endothelial cell proliferation and angiogenic activity [27,28]. Reduced HMGB2 expression has been associated with impaired endothelial tube formation, supporting its potential role in maintaining endothelial function [28]. In experimental ischemic stroke, HMGB2 was reported to promote microglial pro-inflammatory responses, whereas its inhibition reduced brain injury [29]. In this context, our findings suggest that miR-558-mediated suppression of HMGB2 may contribute to impaired endothelial angiogenic function in MMD. However, the specific role of HMGB2 in MMD remains to be established.

Previous studies have identified diverse circulating miRNA signatures associated with MMD using serum, plasma, cerebrospinal fluid, and exosomal fractions, implicating multiple pathways involved in angiogenesis, inflammation, and vascular remodeling [21,22,23,24,25]. However, the overlap among reported miRNA profiles has been limited, reflecting substantial heterogeneity in sample types, analytical platforms, and study designs. In addition, most studies compared patients with MMD only with healthy controls and focused primarily on differential expression or pathway prediction without functional validation of candidate miRNAs.

In contrast, our study incorporated ICAS as a disease control and focused specifically on circulating EV-incorporated miRNAs, which may provide a more disease-relevant view of EV cargo associated with endothelial dysfunction than free-circulating miRNAs. Furthermore, by integrating independent cohort validation, patient-derived iPSC-endothelial cells, and functional target analyses, we identified circulating EV-miR-558 as a biologically relevant regulator of endothelial dysfunction and demonstrated that HMGB2 is a direct target of miR-558. These methodological and mechanistic differences may explain the limited overlap with previously reported miRNA signatures while supporting the relevance of EV-miR-558 as a functionally active EV-associated miRNA in MMD-related endothelial dysfunction.

The limited overlap between the miRNA signatures identified in this study and those reported previously further highlights the importance of sample selection and EV isolation strategies. Differences in biofluids (plasma, serum, or cerebrospinal fluid), miRNA compartments (free-circulating versus EV-incorporated), and comparator groups may substantially influence the detected miRNA profiles [12,22]. In particular, some miRNAs reported in previous studies may reflect nonspecific responses to cerebral ischemia or intracranial arterial stenosis rather than MMD-specific molecular alterations.

In the present study, plasma was selected instead of serum to minimize contamination by platelet-derived EVs generated during clot formation, which account for a substantial proportion of EVs present in serum [30]. Moreover, previous studies have demonstrated distinct miRNA compositions among plasma, EVs, and EV-depleted fractions, indicating that EV-associated miRNAs represent a selective and biologically regulated subset of circulating miRNAs [22,31]. Thus, focusing on EV-incorporated miRNAs may better capture biologically meaningful EV cargo associated with endothelial dysfunction. These observations support the use of circulating EV-derived miRNAs as a more stable and disease-relevant source of molecular information and may explain the identification of EV-miR-558 as a MMD-associated biomarker with functional relevance to endothelial dysfunction.

This study has several limitations. First, the study population was relatively small and, importantly, consisted exclusively of Korean patients with MMD carrying the East Asian founder variant RNF213 p.R4810K. Because the prevalence and spectrum of RNF213 variants differ markedly across ethnic groups—p.R4810K being highly common in East Asian MMD but uncommon in European and other populations—our findings should be regarded as specific to an East Asian, R4810K-enriched genetic background and may not be directly generalizable to patients of other ethnicities or to those harboring different RNF213 variants or wild-type RNF213. This constraint applies both to the mechanistic role of the EV-miR-558–HMGB2 axis and to its potential utility as a circulating biomarker. Accordingly, multicenter studies enrolling ethnically diverse cohorts, as well as MMD patients with different RNF213 genotypes, will be essential to establish the broader biological and clinical relevance of EV-miR-558 in MMD-related endothelial dysfunction. Second, we focused primarily on EV-incorporated miRNAs and did not evaluate EV-depleted plasma or circulating proteomic profiles. Although previous studies suggest that EV-associated miRNAs provide a more stable and biologically relevant source of circulating RNA than whole plasma [32], integrated multi-omic approaches combining EV-miRNAs with protein biomarkers may further improve mechanistic understanding of EV cargo-mediated endothelial alterations in MMD. Third, our functional analyses were performed primarily in HUVECs and validated using patient-derived iPSC-endothelial cells from three independent donors per group, although the small number of lines and donor-to-donor variability limited more extensive functional analyses in this model. Although these models reflect the characteristic angiogenic abnormalities of MMD, the cellular origins of circulating EVs and the contribution of other vascular cell types, including vascular smooth muscle cells, remain to be elucidated. However, direct uptake of patient-derived plasma EVs and EV-mediated transfer of miR-558 to endothelial cells were not experimentally demonstrated in this study. Future studies incorporating cell-type-specific EV profiling and in vivo validation will provide a more comprehensive understanding of EV cargo-mediated vascular remodeling in MMD. Finally, our analysis was limited to symptomatic patients with MMD. Longitudinal studies including asymptomatic, stable, and progressive disease stages are warranted to determine whether circulating EV-miR-558 is associated with disease initiation, progression, or clinical outcomes, and to further establish its utility as a biomarker for disease monitoring and therapeutic response.

## 5. Conclusions

In conclusion, our study identifies circulating EV-miR-558 as a promising candidate biomarker and functional regulator of endothelial dysfunction in MMD. By integrating plasma EV profiling, independent cohort validation, patient-derived iPSC-endothelial cells, and mechanistic analyses, we demonstrate that miR-558 directly suppresses HMGB2 and impairs endothelial angiogenic function. These findings support a potential mechanistic link between circulating EV-miRNA dysregulation and vascular pathology in MMD, supporting EV-miR-558 as a promising candidate biomarker and potential therapeutic target. Further prospective and longitudinal studies involving diverse patient populations are warranted to validate its clinical utility for disease diagnosis, disease monitoring, and therapeutic stratification.

## Figures and Tables

**Table 1:** Baseline characteristics.

	Groups			*p* Value	
	MMD	HC	ICAS	MMD vs. HC	MMD vs. ICAS
Cohorts	*n* = 74	*n* = 49	*n* = 50		
Pool	10	10	10		
Individual	40	39	22		
Validation	24		18		
Age, mean (SD)	46.8 (13.5)	52.2 (9.1)	62.6 (13.7)	*<0.001*	*<0.001*
Female	51 (68.9%)	35 (71.4%)	20 (40.0%)	*NS*	*0.001*
Risk factor					
Hypertension	38 (51.4%)	19 (38.8%)	39 (78.0%)	*NS*	*0.001*
Diabetes	14 (18.9%)	5 (10.2%)	25 (50.0%)	*NS*	*<0.001*
Dyslipidemia	22 (29.7%)	7 (14.3%)	32 (64.0%)	*NS*	*<0.001*
Smoking	5 (6.8%)	5 (10.2%)	17 (34.0%)	*NS*	*<0.001*
*RNF213* variant	74 (100%)	N/A	0 (0%)		
Clinical presentation					
Asymptomatic	5 (6.8%)		0 (0%)		
Transient ischemic attack	25 (33.8%)		8 (16.0%)		
Ischemic stroke	35 (47.3%)		42 (84.0%)		
Intracerebral hemorrhage	9 (12.2%)		0 (0%)		
Medications					
Antiplatelets	64 (86.5%)	0 (0%)	50 (100%)		
Statins	24 (32.4%)	7 (14.3%)	49 (98%)		

MMD, moyamoya disease; HC, healthy control; ICAS, intracranial atherosclerosis; NS, non-significant (*p* > 0.05).

## Data Availability

This study is a part of the clinical trial ‘Moyamoya Disease Biomarkers in Patients with Intracranial Atherosclerotic Stroke’ (URL: https://clinicaltrials.gov (accessed on 18 July 2025). Unique identifier: NCT02074111). Data supporting the findings of this study are available from the corresponding author upon request.

## Supplementary Materials

The following supporting information can be downloaded at https://www.mdpi.com/article/10.3390/cells15161456/s1, Supplementary data are available in the Supplementary Material. Figure S1. Characteristics of plasma extracellular vesicles (EVs). (A) Process for the isolation of EVs. (B) Nanoparticle Tracking Analysis was used to calculate size distribution of EVs. (C) Electron microscopy and cryo-electron microscopy were used to determine the size and lipid bilayers of purified EVs. (D) Western blot analysis of extracellular vesicle markers. The Western blot images shown are cropped, and the original uncropped blots are no longer available. Figure S2. Effects of miR-623 and its target. (A) Representative phase-contrast images showing the typical cobblestone morphology of HUVECs transfected with miR-623. (B) Wound healing assay demonstrating the effect of miR-623 on the migratory capacity of HUVECs. Images were captured at 0 and 7 h after scratch induction. The bar graph shows the quantification of wound closure (%). (C) Western blot was performed on HUVEC lysates using antibodies against CAV-1 and GAPDH. Band intensities were quantified by densitometric analysis and normalized to GAPDH. The Western blot images shown are cropped, and the original uncropped blots are no longer available. (D) Schematic of CAV1 transcript and reporter construct (upper panel). Luciferase activity in HEK293 co-transfected with wild-type or mutant reporter with miR-623 mimic, normalized to Renilla luciferase activity. Data are presented as mean ± SD from three independent experiments (*n* = 3). * *p* < 0.05. *** *p* < 0.001. Figure S3. Effects of miR-548b-3p and its targets. (A–C) HUVECs were transfected with control miRNA, miRNA mimic or miRNA inhibitor for tube formation, cell proliferation, cell migration assay and western blot. (A) Representative images and quantification results of tube formation (upper panels) and proliferation assays (lower panels). (B) Representative images and quantification results of migration assay. (C) Western blot was performed on HUVEC lysates using antibodies against MTA2 and GAPDH. Band intensities were quantified by densitometric analysis and normalized to GAPDH. The Western blot images shown are cropped, and the original uncropped blots are no longer available. (D) Schematic of MTA2 transcript and reporter construct (upper panel). Luciferase activity in HEK293 co-transfected with wild-type (WT) or mutant (MT) reporter with miR-548b mimic, normalized to Renilla luciferase activity. Data are presented as mean ± SD from three independent experiments (*n* = 3). * *p* < 0.05. Figure S4. Characterization of healthy control (HC) and MMD patient-derived iPSCs. (A) Representative G-banded karyotype analyses showing normal chromosomal patterns (46, XX or 46, XY) in iPSCs derived from healthy individuals and MMD patients. Metaphase spreads and corresponding karyograms are displayed. (B) Flow cytometry showing high expression (>90%) of the pluripotency surface marker SSEA-4 in iPSCs from both HC and MMD donors. (C) Immunocytochemistry images confirming strong expression of the pluripotency markers OCT4 (red, nuclear) and SSEA4 (green, cytoplasmic) in iPSCs. Nuclei were counterstained with DAPI (blue). Merged and single-channel images are shown. Figure S5. Flow cytometric analysis of endothelial markers in HC and MMD patient-derived iPSC-ECs. (A) Representative dot plots of CD31 and CD144 co-expression in HC and MMD patient-derived iPSC-ECs. The numbers indicate the percentage of CD31 and CD144 double positive cells. (B) Percentage of endothelial cell and hematopoietic markers in HC and MMD patient derived iPSC-ECs. Data are represented as mean ± SD. Dashed line indicates criteria for successful endothelial differentiation.

## Abbreviations

AUC, area under the curve; EV, extracellular vesicle; HUVEC, human umbilical vein endothelial cell; ICAS, intracranial atherosclerotic stenosis; iPSC-EC, induced pluripotent stem cell-derived endothelial cell; MMD, moyamoya disease; NTA, nanoparticle tracking analysis; PBMC, peripheral blood mononuclear cell; qRT-PCR, quantitative reverse-transcription PCR; ROC, receiver operating characteristic; TEM, transmission electron microscopy.

## References

[1] Ihara M., Yamamoto Y., Hattori Y., Liu W., Kobayashi H., Ishiyama H., Yoshimoto T., Miyawaki S., Clausen T., Bang O.Y. (2022). Moyamoya disease: Diagnosis and interventions. Lancet Neurol..

[2] Bang O.Y., Chung J.W., Kim S.J., Oh M.J., Kim S.Y., Cho Y.H., Cha J., Yeon J.Y., Kim K.H., Kim G.M. (2016). Caveolin-1, Ring finger protein 213, and endothelial function in Moyamoya disease. Int. J. Stroke.

[3] Chung J.W., Kim D.H., Oh M.J., Cho Y.H., Kim E.H., Moon G.J., Ki C.S., Cha J., Kim K.H., Jeon P. (2018). Cav-1 (Caveolin-1) and Arterial Remodeling in Adult Moyamoya Disease. Stroke.

[4] Ikeuchi Y., Kitayama J., Sahara N., Okata T., Miyake N., Matsumoto N., Kitazono T., Ago T. (2022). Filamin A Variant as a Possible Second-Hit Gene Promoting Moyamoya Disease-like Vascular Formation Associated with RNF213 p.R4810K Variant. Neurol. Genet..

[5] Koizumi A., Kobayashi H., Hitomi T., Harada K.H., Habu T., Youssefian S. (2016). A new horizon of moyamoya disease and associated health risks explored through RNF213. Environ. Health Prev. Med..

[6] Ryoo S., Cha J., Kim S.J., Choi J.W., Ki C.S., Kim K.H., Jeon P., Kim J.S., Hong S.C., Bang O.Y. (2014). High-resolution magnetic resonance wall imaging findings of moyamoya disease. Stroke.

[7] Fang Y.C., Wei L.F., Hu C.J., Tu Y.K. (2021). Pathological Circulating Factors in Moyamoya Disease. Int. J. Mol. Sci..

[8] Derdeyn C.P., Chimowitz M.I., Lynn M.J., Fiorella D., Turan T.N., Janis L.S., Montgomery J., Nizam A., Lane B.F., Lutsep H.L. (2014). Aggressive medical treatment with or without stenting in high-risk patients with intracranial artery stenosis (SAMMPRIS): The final results of a randomised trial. Lancet.

[9] Natarajan S.K., Karmon Y., Tawk R.G., Hauck E.F., Hopkins L.N., Siddiqui A.H., Levy E.I. (2011). Endovascular treatment of patients with intracranial stenosis with moyamoya-type collaterals. J. Neurointerv. Surg..

[10] Khan N., Dodd R., Marks M.P., Bell-Stephens T., Vavao J., Steinberg G.K. (2011). Failure of primary percutaneous angioplasty and stenting in the prevention of ischemia in Moyamoya angiopathy. Cerebrovasc. Dis..

[11] Chung J.W., Kim S.J., Bang O.Y., Kim K.H., Ki C.S., Jeon P., Yeon J.Y., Kim J.S., Hong S.C., Shin H.J. (2016). Determinants of Basal Collaterals in Moyamoya Disease: Clinical and Genetic Factors. Eur. Neurol..

[12] Tiedt S., Dichgans M. (2018). Role of Non-Coding RNAs in Stroke. Stroke.

[13] Slomka A., Kornek M., Cho W.C. (2022). Small Extracellular Vesicles and Their Involvement in Cancer Resistance: An Up-to-Date Review. Cells.

[14] van Kralingen J.C., McFall A., Ord E.N.J., Coyle T.F., Bissett M., McClure J.D., McCabe C., Macrae I.M., Dawson J., Work L.M. (2019). Altered Extracellular Vesicle MicroRNA Expression in Ischemic Stroke and Small Vessel Disease. Transl. Stroke Res..

[15] Niu M., Li H., Li X., Yan X., Ma A., Pan X., Zhu X. (2021). Circulating Exosomal miRNAs as Novel Biomarkers Perform Superior Diagnostic Efficiency Compared with Plasma miRNAs for Large-Artery Atherosclerosis Stroke. Front. Pharmacol..

[16] Miyawaki S., Imai H., Takayanagi S., Mukasa A., Nakatomi H., Saito N. (2012). Identification of a genetic variant common to moyamoya disease and intracranial major artery stenosis/occlusion. Stroke.

[17] Bang O.Y., Chung J.W., Cha J., Lee M.J., Yeon J.Y., Ki C.S., Jeon P., Kim J.S., Hong S.C. (2016). A Polymorphism in RNF213 Is a Susceptibility Gene for Intracranial Atherosclerosis. PLoS ONE.

[18] Smith A.S.T., Kim J.H., Chun C., Gharai A., Moon H.W., Kim E.Y., Nam S.H., Ha N., Song J.Y., Chung K.W. (2022). HDAC6 Inhibition Corrects Electrophysiological and Axonal Transport Deficits in a Human Stem Cell-Based Model of Charcot-Marie-Tooth Disease (Type 2D). Adv. Biol..

[19] Tang L., Su J., Liang P. (2017). Modeling cadmium-induced endothelial toxicity using human pluripotent stem cell-derived endothelial cells. Sci. Rep..

[20] Ota S., Yokoyama K., Kanamori F., Mamiya T., Uda K., Araki Y., Wakabayashi T., Yoshikawa K., Saito R. (2023). Moyamoya disease-specific extracellular vesicle-derived microRNAs in the cerebrospinal fluid revealed by comprehensive expression analysis through microRNA sequencing. Acta Neurochir..

[21] Dai D., Lu Q., Huang Q., Yang P., Hong B., Xu Y., Zhao W., Liu J., Li Q. (2014). Serum miRNA signature in Moyamoya disease. PLoS ONE.

[22] Lee M.J., Fallen S., Zhou Y., Baxter D., Scherler K., Kuo M.F., Wang K. (2019). The Impact of Moyamoya Disease and RNF213 Mutations on the Spectrum of Plasma Protein and MicroRNA. J. Clin. Med..

[23] Uchino H., Ito M., Kazumata K., Hama Y., Hamauchi S., Terasaka S., Sasaki H., Houkin K. (2018). Circulating miRNome profiling in Moyamoya disease-discordant monozygotic twins and endothelial microRNA expression analysis using iPS cell line. BMC Med. Genom..

[24] Wang G., Wen Y., Faleti O.D., Zhao Q., Liu J., Zhang G., Li M., Qi S., Feng W., Lyu X. (2020). A Panel of Exosome-Derived miRNAs of Cerebrospinal Fluid for the Diagnosis of Moyamoya Disease. Front. Neurosci..

[25] Zhao S., Gong Z., Zhang J., Xu X., Liu P., Guan W., Jing L., Peng T., Teng J., Jia Y. (2015). Elevated Serum MicroRNA Let-7c in Moyamoya Disease. J. Stroke Cerebrovasc. Dis..

[26] Gong M., Yu B., Wang J., Wang Y., Liu M., Paul C., Millard R.W., Xiao D.S., Ashraf M., Xu M. (2017). Mesenchymal stem cells release exosomes that transfer miRNAs to endothelial cells and promote angiogenesis. Oncotarget.

[27] Ueda T., Yoshida M. (2010). HMGB proteins and transcriptional regulation. Biochim. Biophys. Acta.

[28] Jo H.R., Jeong J.H. (2022). MicroRNA-Mediated Downregulation of HMGB2 Contributes to Cellular Senescence in Microvascular Endothelial Cells. Cells.

[29] Wu J., Wu Z., He A., Zhang T., Zhang P., Jin J., Li S., Li G., Li X., Liang S. (2021). Genome-Wide Screen and Validation of Microglia Pro-Inflammatory Mediators in Stroke. Aging Dis..

[30] Witwer K.W., Buzas E.I., Bemis L.T., Bora A., Lasser C., Lotvall J., Nolte-’t Hoen E.N., Piper M.G., Sivaraman S., Skog J. (2013). Standardization of sample collection, isolation and analysis methods in extracellular vesicle research. J. Extracell. Vesicles.

[31] Ramanathan S., Shenoda B.B., Lin Z., Alexander G.M., Huppert A., Sacan A., Ajit S.K. (2019). Inflammation potentiates miR-939 expression and packaging into small extracellular vesicles. J. Extracell. Vesicles.

[32] Endzelins E., Berger A., Melne V., Bajo-Santos C., Sobolevska K., Abols A., Rodriguez M., Santare D., Rudnickiha A., Lietuvietis V. (2017). Detection of circulating miRNAs: Comparative analysis of extracellular vesicle-incorporated miRNAs and cell-free miRNAs in whole plasma of prostate cancer patients. BMC Cancer.

