# Supplementary material for: Extracellular Vesicle miR-558 Regulates Endothelial Function Through HMGB2 in Adult Moyamoya Disease
Source: Cells. 2026 Aug 13;15(16):1456. doi: 10.3390/cells15161456 (PMC13510406; doi:10.3390/cells15161456)

1. Isolation and characterization of plasma EVs

NTA showed mean and mode sizes of 197.0 ± 6.3 nm and 144.5 ± 18.9 nm for the EV (microvesicle) and particle-to-protein ratio was 2.0 × 10^8 particles/μg protein (Figure S1B). TEM and cryo-TEM analyses revealed that the **EVs** had a round shape with a high electron density structure (**Figure S1C**). The EVs were positive for EV markers such as CD63, CD81, Flotillin-1, and TSG101 and negative for calnexin, an EV negative marker (**Figure S1D**).

Figure S1. Characteristics of plasma extracellular vesicles (EVs). (A) Process for the isolation of EVs. (B) Nanoparticle Tracking Analysis was used to calculate size distribution of EVs. (C) Electron microscopy and cryo-electron microscopy were used to determine the size and lipid bilayers of purified EVs. (D) Western blot analysis of extracellular vesicle markers. The Western blot images shown are cropped, and the original uncropped blots are no longer available.

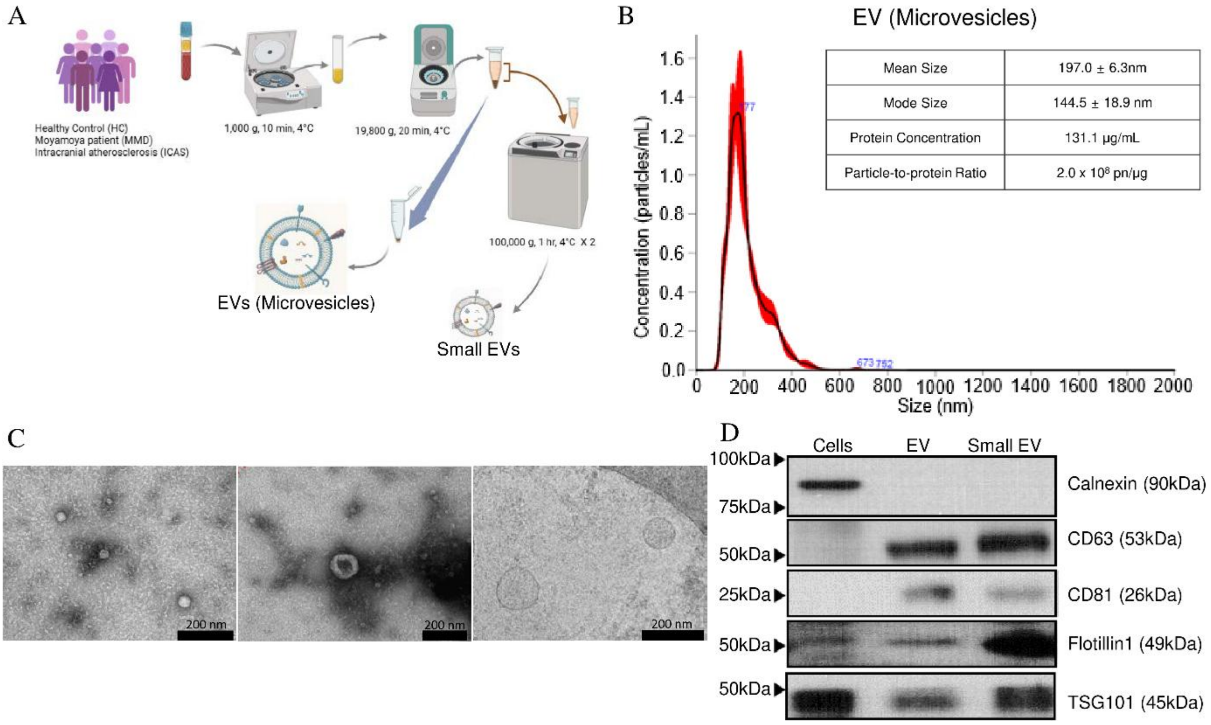

2. Effects of miR-623 and miR-548b-3p on endothelial function and identification of their target genes.

To elucidate the effects of miR-623 (**Figure S2**) and miR-548b-3p (**Figure S3**) on endothelial cell function, HUVECs were transfected with miRNA mimics and inhibitors. Transfection with the miR-623 mimic inhibited endothelial function, suppressing both tube formation and cell proliferation. In contrast, cell migration was not significantly altered by the miR-623 mimic. Conversely, the miR-623 inhibitor enhanced both tube formation and cell proliferation. Regarding miR-548b-3p, transfection with its mimic inhibited tube formation, but did not significantly affect cell proliferation or migration. Conversely, the miR-548b-3p inhibitor significantly enhanced cell proliferation, but did not affect tube formation or migration.

Western blot analysis was performed to investigate the effect of these miRNAs on their predicted target proteins. Transfection with the miR-623 mimic reduced the protein levels of its predicted target, Caveolin-1 (CAV1), normalized to GAPDH. Similarly, transfection with the miR-548b-3p mimic reduced the protein levels of its predicted target, MTA2. To validate these predicted targets, luciferase reporter assays were performed using constructs containing the 3’UTR regions of the predicted target genes. Co-transfection of a wild-type CAV1 3’UTR reporter construct with the miR-623 mimic resulted in a significant reduction in luciferase activity, and this repression was abolished by mutation of the predicted binding site within the 3’UTR. In contrast, co-transfection of a wild-type MTA2 3’UTR reporter construct with the miR-548b-3p mimic did not reduce luciferase activity, indicating that MTA2 was not confirmed as a direct target of miR-548b-3p.

Figure S2. Effects of miR-623 and its target. (A) Representative phase-contrast images showing the typical cobblestone morphology of HUVECs transfected with miR-623. (B) Wound healing assay demonstrating the effect of miR-623 on the migratory capacity of HUVECs. Images were captured at 0 and 7 hours after scratch induction. The bar graph shows the quantification of wound closure (%). (C) Western blot was performed on HUVEC lysates using antibodies against CAV-1 and GAPDH. Band intensities were quantified by densitometric analysis and normalized to GAPDH. The Western blot images shown are cropped, and the original uncropped blots are no longer available. (D) Schematic of CAV1 transcript and reporter construct (upper panel). Luciferase activity in HEK293 co-transfected with wild-type or mutant reporter with miR-623 mimic, normalized to Renilla luciferase activity. Data are presented as mean ± SD **from three independent experiments (n = 3).** *P<0.05. ***P<0.001.

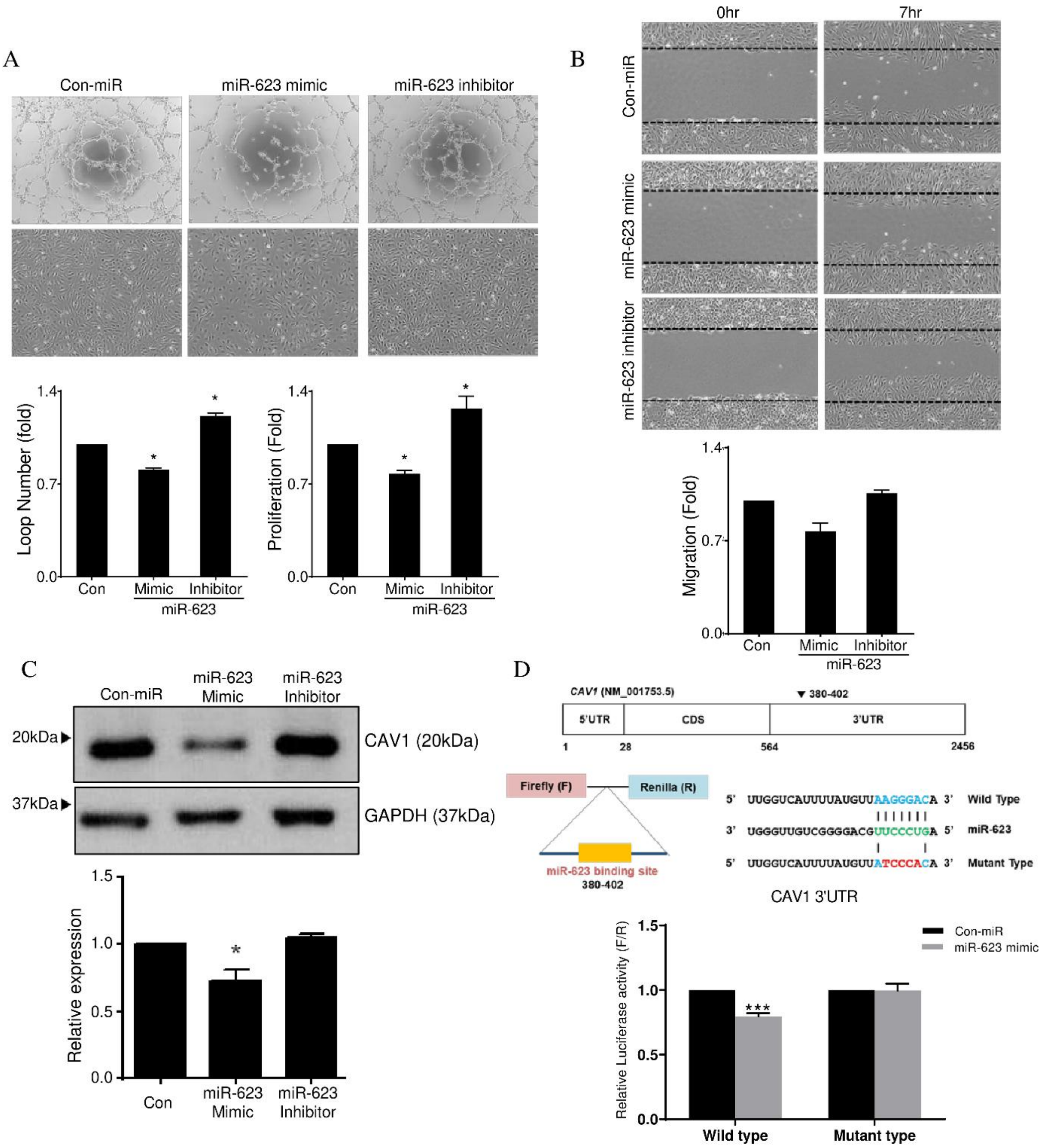
A
Con-miR
miR-623 mimic
miR-623 inhibitor
Loop Number (fold)
Proliferation (Fold)
Con
Mimic
Inhibitor
miR-623
B
0hr
7hr
Con-miR
miR-623 mimic
miR-623 inhibitor
Migration (Fold)
C
Con-miR
miR-623 Mimic
miR-623 Inhibitor
20kDa
37kDa
CAV1 (20kDa)
GAPDH (37kDa)
Relative expression
D
CAV1 (NM_001753.5)
380-402
5'UTR
CDS
3'UTR
1
28
564
2456
Firefly (F)
Renilla (R)
miR-623 binding site
380-402
5' UUGGUCAUUUUAUGUUAAGGGACA 3' Wild Type
3' UGGGUUGUCGGGGACGUUCCCUGA 5' miR-623
5' UUGGUCAUUUUAUGUUATCCCACA 3' Mutant Type
CAV1 3'UTR
Relative Luciferase activity (F/R)
Con-miR
miR-623 mimic
Wild type
Mutant type

Figure S3. Effects of miR-548b-3p and its targets. (A-C) HUVECs were transfected with control miRNA, miRNA mimic or miRNA inhibitor for tube formation, cell proliferation, cell migration assay and western blot. (A) Representative images and quantification results of tube formation (upper panels) and proliferation assays (lower panels). (B) Representative images and quantification results of migration assay. (C) Western blot was performed on HUVEC lysates using antibodies against MTA2 and GAPDH. Band intensities were quantified by densitometric analysis and normalized to GAPDH. The Western blot images shown are cropped, and the original uncropped blots are no longer available. (D) Schematic of MTA2 transcript and reporter construct (upper panel). Luciferase activity in HEK293 co-transfected with wild-type (WT) or mutant (MT) reporter with miR-548b mimic, normalized to Renilla luciferase activity. Data are presented as mean ± SD **from three independent experiments (n = 3).** *$P<0.05$.

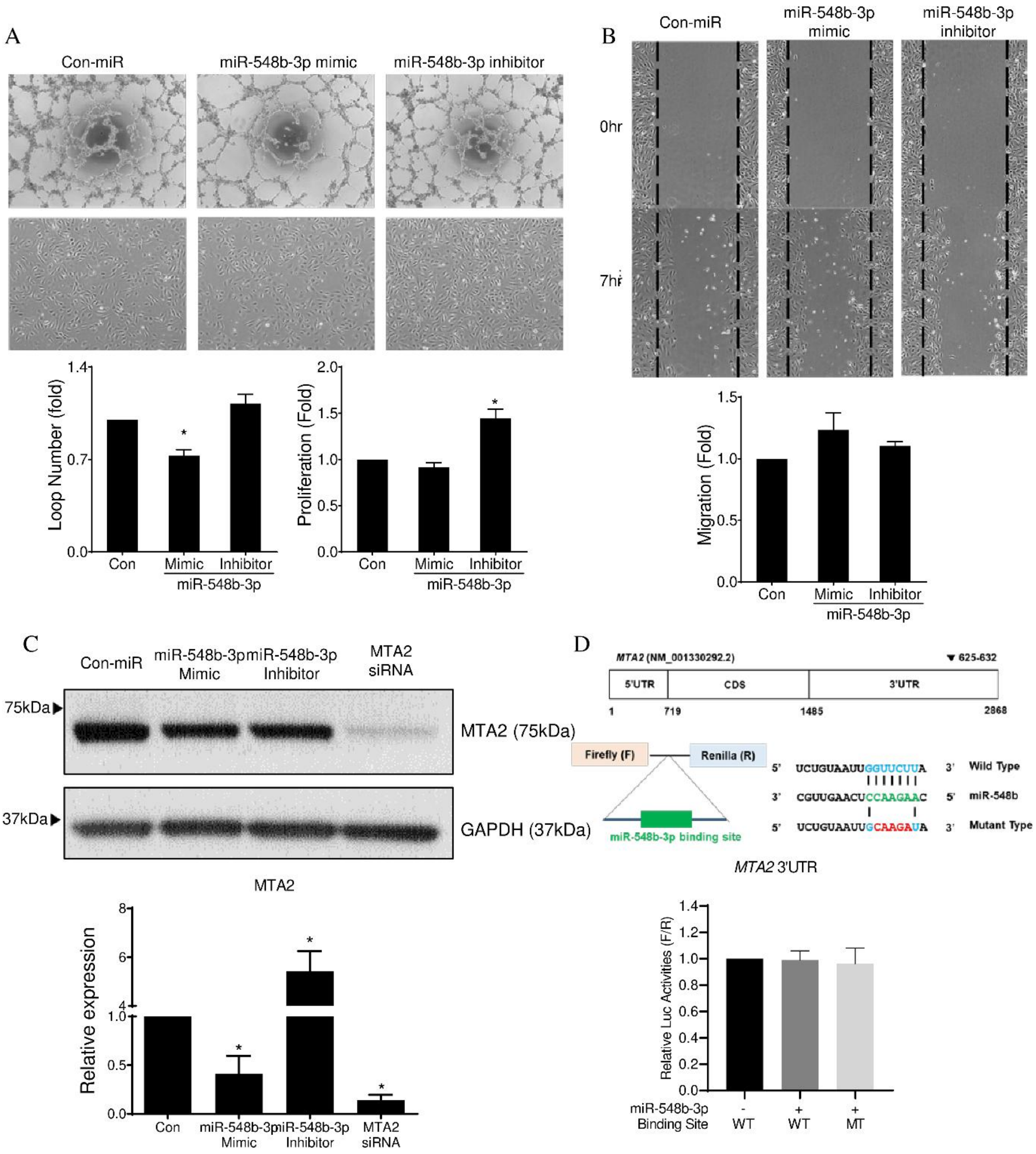

A
Con-miR
miR-548b-3p mimic
miR-548b-3p inhibitor
Loop Number (fold)
1.4
0.7
0.0
Con
Mimic
Inhibitor
miR-548b-3p
Proliferation (Fold)
2.0
1.5
1.0
0.5
0.0
Con
Mimic
Inhibitor
miR-548b-3p
B
Con-miR
miR-548b-3p mimic
miR-548b-3p inhibitor
0hr
7hr
Migration (Fold)
1.5
1.0
0.5
0.0
Con
Mimic
Inhibitor
miR-548b-3p
C
Con-miR
miR-548b-3p Mimic
miR-548b-3p Inhibitor
MTA2 siRNA
75kDa
MTA2 (75kDa)
37kDa
GAPDH (37kDa)
MTA2
Relative expression
8
6
4
1.0
0.5
0.0
Con
miR-548b-3p Mimic
miR-548b-3p Inhibitor
MTA2 siRNA
D
MTA2 (NM_001330292.2)
625-632
5'UTR
CDS
3'UTR
1
719
1485
2868
Firefly (F)
Renilla (R)
miR-548b-3p binding site
5' UCUGUAAUUGGUUCUUA 3' Wild Type
3' CGUUGAACUCCAAGAAC 5' miR-548b
5' UCUGUAAUUGCAAGAUA 3' Mutant Type
MTA2 3'UTR
Relative Luc Activities (F/R)
1.4
1.2
1.0
0.8
0.6
0.4
0.2
0.0
miR-548b-3p
Binding Site
-
WT
+
WT
+
MT

3. Validation and Characterization of iPSCs

Generation of Patient-specific Induced Pluripotent Stem Cells (iPSCs)

Peripheral blood mononuclear cells (PBMCs) were isolated from whole blood obtained from patients using SepMate™ PBMC Isolation Tubes (Cat. #86450, STEMCELL Technologies, Vancouver, BC, Canada) in combination with Ficoll-Paque PLUS (Cat. No. GE17-1440, GE Healthcare, Chicago, IL), following the manufacturer's instructions. Isolated PBMCs were reprogrammed into induced pluripotent stem cells (iPSCs) utilizing the CytoTune®-iPS 2.0 Sendai Reprogramming Kit (Cat. No. A34546; Thermo Fisher Scientific, Waltham, MA), according to the manufacturer's protocol. The reprogramming process was carried out over approximately 60 days. During this period, transgene-free iPSC colonies displaying typical pluripotent stem cell morphology were manually selected, clonally expanded, and subjected to validation.

Karyotyping

G-banded karyotype analysis was performed to assess potential chromosomal abnormalities in the iPSCs. Cells were treated with KaryoMAX Colcemid solution (0.1 μg/mL; 15212012, Thermo Fisher Scientific, Waltham, MA) for 2 hours, harvested by trypsinization using TrypLE Express (12605010, Thermo Fisher Scientific, Waltham, MA), and subjected to hypotonic treatment with 75 mM KCl for 30 min at 37 °C. After fixation with a 3:1 methanol:glacial acetic acid solution, cell suspensions were dropped onto glass slides and air-dried. Slides were then processed according to the standard G-banding protocol. Karyotype analysis was outsourced to the Korea Research Animal Cytogenetics Laboratory (KRACH; www.krach.co.kr) in Gwangjin-gu, Seoul, Republic of Korea.

Flow Cytometric Analysis of SSEA-4 Expression

Undifferentiated iPSCs were dissociated into single-cell suspensions during passaging. Cells were washed with phosphate-buffered saline (PBS) containing 1% fetal bovine serum (FBS) and subsequently incubated for 20 min at 4 °C with PE-conjugated mouse anti-SSEA-4 antibody (BD Biosciences, San Jose, CA), protected from light. Unbound antibody was removed through additional washes with PBS + 1% FBS. The labeled cells were immediately analyzed on a BD FACSVerse flow cytometer (BD Biosciences, San Jose, CA) and fluorescence data were processed using BD FACSuite software to quantify SSEA4-positive cell populations.

IPSCs immunocytochemistry

To evaluate pluripotency, induced pluripotent stem cells (iPSCs) were fixed with 4% paraformaldehyde (Sigma-Aldrich, St. Louis, MO) for 20 min at room temperature. Following fixation, cells were permeabilized and blocked in phosphate-buffered saline (PBS) containing 0.1% Triton X-100 (Sigma-Aldrich, St. Louis, MO) and 10% goat serum (Thermo Fisher Scientific, Waltham, MA) for 1 h at room temperature. Primary antibodies against OCT4 (1:200; Millipore, Billerica, MA) and SSEA4 (1:200; sc-21704; Santa Cruz Biotechnology, Dallas, TX) were applied overnight at 4 °C. Fluorophore-conjugated secondary antibodies (Jackson ImmunoResearch Laboratories, West Grove, PA) were used to visualize primary antibody binding. Nuclei were counterstained with 4′,6-diamidino-2-phenylindole (DAPI; 1:1000; Sigma-Aldrich, St. Louis, MO). Images were acquired using a laser-scanning confocal microscope (LSM 8 Exciter; Carl Zeiss, Oberkochen, Germany).

Figure S4. Characterization of healthy control (HC) and MMD patient-derived iPSCs. (A) Representative G-banded karyotype analyses showing normal chromosomal patterns (46, XX or 46, XY) in iPSCs derived from healthy individuals and MMD patients. Metaphase spreads and corresponding karyograms are displayed. (B) Flow cytometry showing high expression (>90%) of the pluripotency surface marker SSEA-4 in iPSCs from both HC and MMD donors. (C) Immunocytochemistry images confirming strong expression of the pluripotency markers OCT4 (red, nuclear) and SSEA4 (green, cytoplasmic) in iPSCs. Nuclei were counterstained with DAPI (blue). Merged and single-channel images are shown.

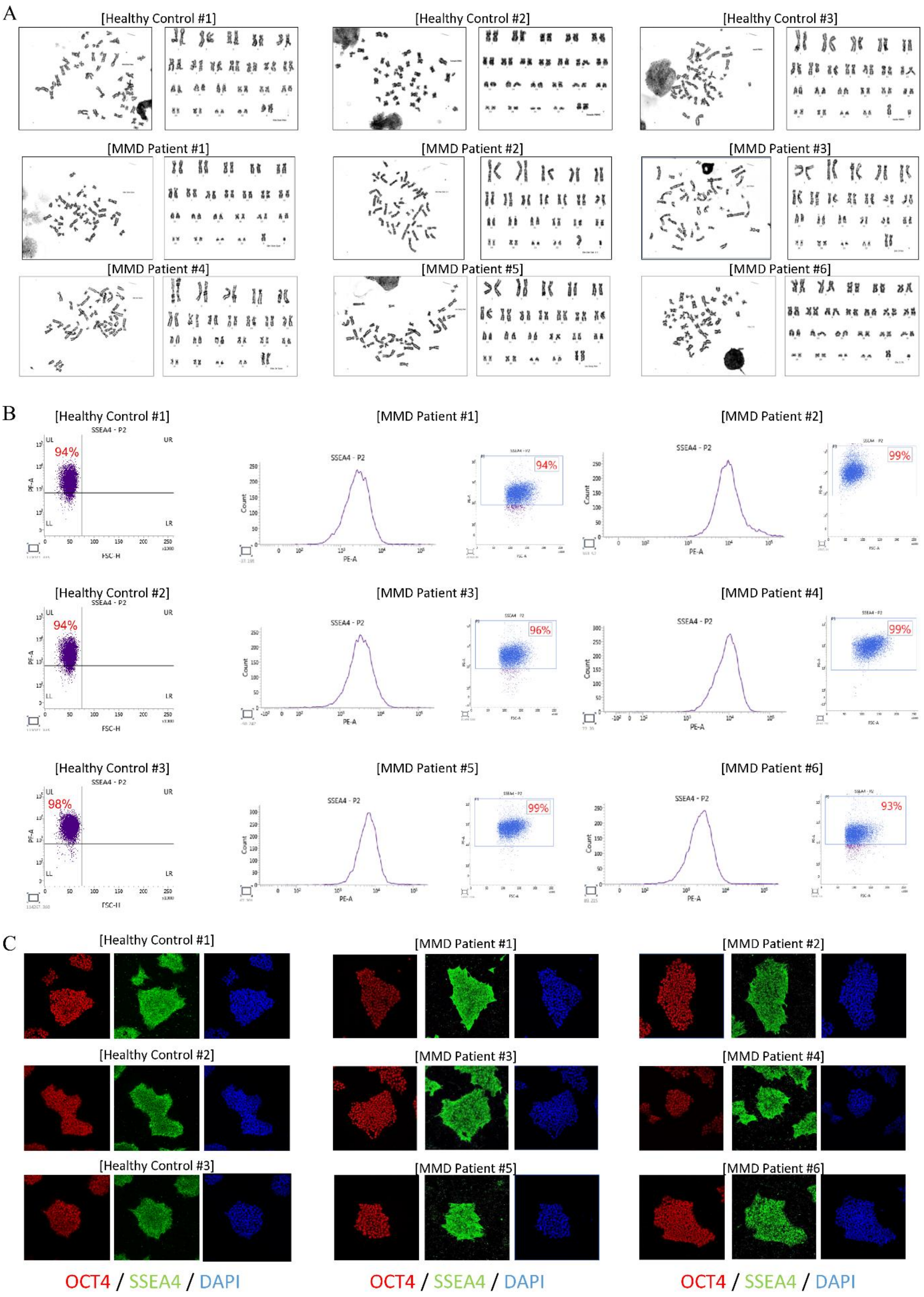
A
[Healthy Control #1]
[Healthy Control #2]
[Healthy Control #3]
[MMD Patient #1]
[MMD Patient #2]
[MMD Patient #3]
[MMD Patient #4]
[MMD Patient #5]
[MMD Patient #6]
B
[Healthy Control #1]
SSEA4 - P2
94%
[MMD Patient #1]
SSEA4 - P2
94%
[MMD Patient #2]
SSEA4 - P2
99%
[Healthy Control #2]
SSEA4 - P2
94%
[MMD Patient #3]
SSEA4 - P2
96%
[MMD Patient #4]
SSEA4 - P2
99%
[Healthy Control #3]
SSEA4 - P2
98%
[MMD Patient #5]
SSEA4 - P2
99%
[MMD Patient #6]
SSEA4 - P2
93%
PE-A
Count
FSC-H
C
[Healthy Control #1]
[MMD Patient #1]
[MMD Patient #2]
[Healthy Control #2]
[MMD Patient #3]
[MMD Patient #4]
[Healthy Control #3]
[MMD Patient #5]
[MMD Patient #6]
OCT4 / SSEA4 / DAPI
OCT4 / SSEA4 / DAPI
OCT4 / SSEA4 / DAPI

Figure S5. Flow cytometric analysis of endothelial markers in HC and MMD patient-derived iPSC-ECs. (A) Representative dot plots of CD31 and CD144 co-expression in HC and MMD patient-derived iPSC-ECs. The numbers indicate the percentage of CD31 and CD144 double positive cells. (B) Percentage of endothelial cell and hematopoietic markers in HC and MMD patient derived iPSC-ECs. Data are represented as mean ± SD. Dashed line indicates criteria for successful endothelial differentiation.

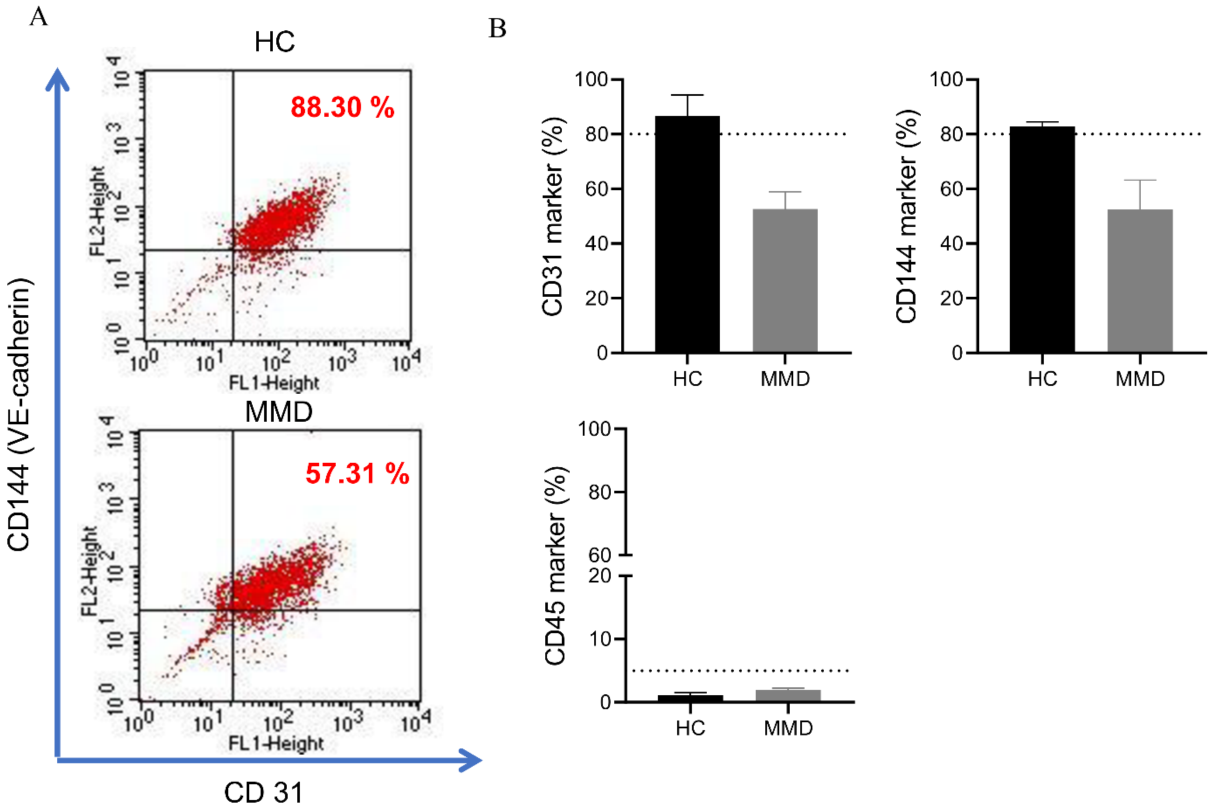

Supplement: Supplementary file 1 [file cells-15-01456-s001.zip › cells-4452703-supplementary.pdf]
